# Mono- and Disamarium Azacryptand Complexes: A Platform for Cooperative Rare-Earth Metal Chemistry

**DOI:** 10.1021/acs.inorgchem.1c03989

**Published:** 2022-03-28

**Authors:** Johanna M. Uher, Matthias R. Steiner, Johann A. Hlina

**Affiliations:** Institute of Inorganic Chemistry, Graz University of Technology, Stremayrgasse 9, 8010 Graz, Austria

## Abstract

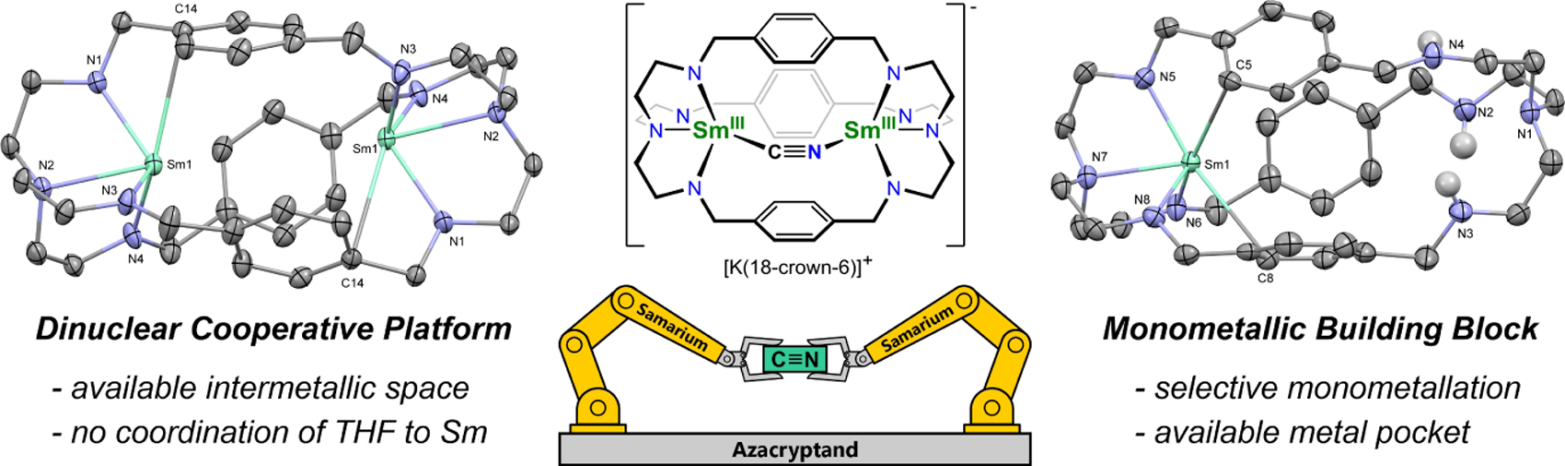

Mono- (H_3_LSm) and disamarium complexes (LSm_2_) were prepared by reaction of the azacryptand N[(CH_2_)_2_NHCH_2_-*p*-C_6_H_4_CH_2_NH(CH_2_)_2_]_3_N (H_6_L) with 1 or 2 equiv of Sm[N(SiMe_3_)_2_]_3_, respectively. The disamarium complex features free coordination sites on both metal centers available for bridging ligands shielded by phenylenes from tetrahydrofuran (THF) coordination. The reaction of LSm_2_ with KCN and 18-crown-6 yielded the adduct [LSm_2_-μ-η^1^:η^1^-CN][K(18-crown-6)(THF)_2_] featuring a bridging cyanide. The complexes were characterized by crystallography, electrochemical analysis, NMR, and optical spectroscopy, and the effective magnetic moments were determined via the Evans method.

## Abstract 2

First examples of rare-earth metal azacryptand complexes are presented in the form of mono- and dinuclear samarium compounds, which can be prepared selectively. The bridging phenylenes of the azacryptand ligand prevent THF coordination to the samarium ions and provide available binding sites within the complexes. We investigated cyanide coordination using the disamarium complex to demonstrate the utility of the vacant intermetallic space for binding small molecules.

## Introduction

The organometallic chemistry of f-block metals exhibits numerous examples in which two monometallic complexes engage in cooperative activation of substrate molecules. Prominent examples in the context of small inert molecules involve reductive coupling of carbon monoxide,^1−6^ dinitrogen reduction,^7−9^ and sulfur dioxide coordination.^10^ Among the rare-earth metals, samarium is a key player in small molecule activation. Typically, two monometallic complexes react with the substrate and form a dinuclear complex in which the two metal ions are bridged by the converted substrate. Using such reactions as a basis for the development of catalytic processes, it appears feasible to utilize ligand frameworks, which already provide a link between the two metal centers. In f-metal chemistry, the number of complexes featuring such an architecture is limited and macrocyclic ligands are most prominent. Here, the so-called Pacman ligands have proven valuable in studying the cooperative chemistry of f-metal ions. The groups of Arnold and Love have reported examples exploring the cooperative interaction of f-block metals with uranium at the center of attention.^11−14^ Macrocyclic azacryptands have been introduced by Lehn and co-workers and the derivatives in which the two tris(2-aminoethyl)amine (TREN) moieties are bridged with phenylenes are ideal ligand frameworks to study cooperative reactivity of dinuclear complexes.^15^ Previously, investigations of such azacryptands focussed on late transition metals and include examples of cooperative interaction such as catalytic carbon dioxide conversion using a dinickel complex.^16^ TREN-based ligands have successfully demonstrated their utility in f-metal chemistry already as exemplified by complexes featuring unusual moieties such as the terminal nitride^17^ or oxide^18^ as well as engaging in reductive carbon monoxide coupling^19^ and metal–metal bonding.^20^ Here, we present samarium compounds as the first examples of mono- and dinuclear f-metal azacryptand complexes.

## Results and Discussion

### Synthesis and Characterization

**Scheme 1:**
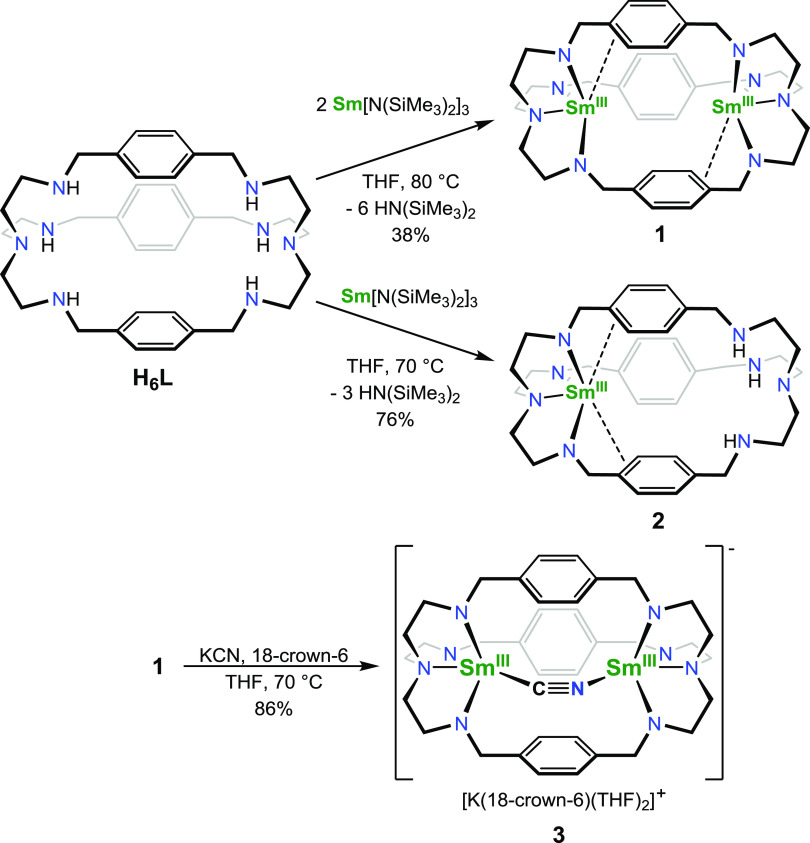
Preparation of the Azacryptand Disamarium (**1**) and Monosamarium (**2**) Complexes as well as the Synthesis of the Azacryptand Disamarium Complex Cyanide Adduct [LSm_2_-μ-η^1^:η^1^-CN][K(18-Crown-6)(THF)_2_] (**3**)

The disamarium azacryptand complex LSm_2_, **1**, was prepared by heating the azacryptand ligand H_6_L (H_6_L = N[(CH_2_)_2_NHCH_2_-*p*-C_6_H_4_CH_2_NH(CH_2_)_2_]_3_N) with 2 equiv of Sm[N(SiMe_3_)_2_]_3_ to 80 °C in tetrahydrofuran (THF) (Scheme 1). During the reaction, **1** precipitated in the form of a yellow powder, which was subsequently isolated in 38% yield. The solubility of **1** is low in DME, toluene, and benzene, and **1** is insoluble in pentane and diethyl ether. Along with the dinuclear samarium complex **1**, we also observed the mononuclear intermediate H_3_LSm, **2**, as a side-product of the reaction, which precipitated along with **1**. Multiple washings of the crude product with THF were done to remove **2**. The washing also accounts for the loss of **1** and its low isolated yield.

**Figure 1:**
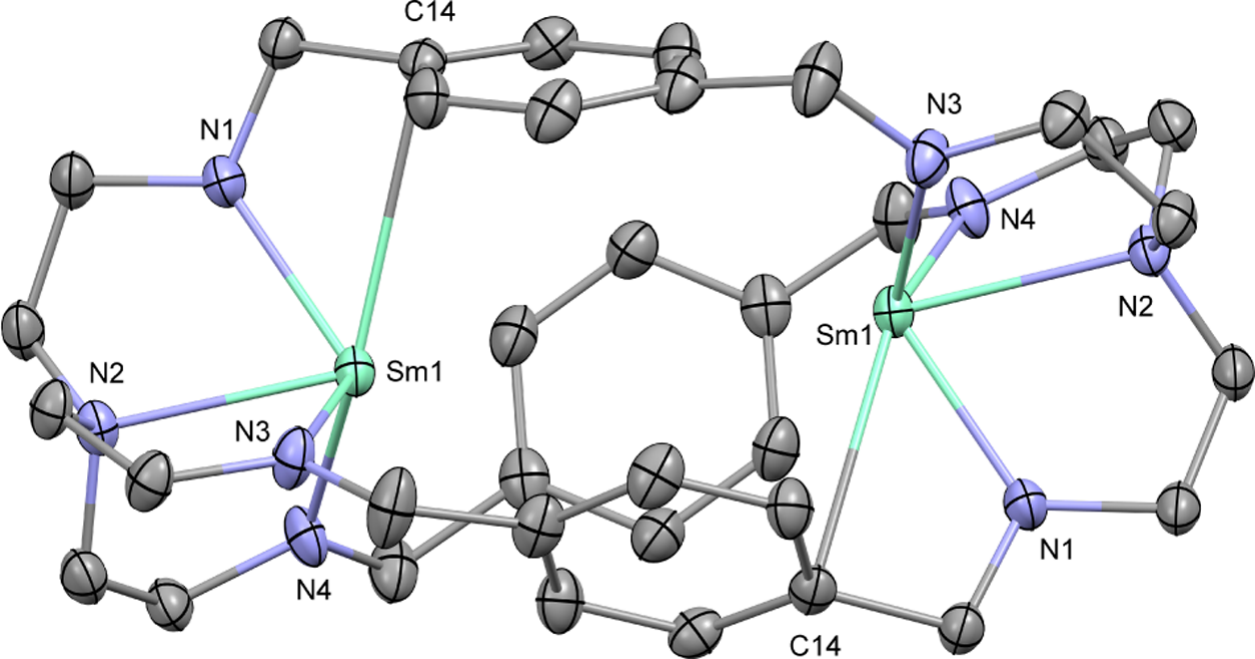
Molecular structure of **1**. Hydrogen atoms are omitted for clarity. Thermal ellipsoids drawn at 50% probability. Selected distances (Å) and angles (deg): Sm1–N1: 2.247(4), Sm1–N2: 2.514(4), Sm1–N3: 2.260(5), Sm1–N4: 2.244(5), Sm1–C14: 3.056(5), Sm1···Sm1′: 5.1758(9), N1–Sm1–N2: 68.6(1), N2–Sm1–N3: 69.7(1), and N2–Sm1–N4: 70.2(1).

Single-crystal X-ray diffraction analysis of a yellow crystal of **1** confirmed the bimetallic structure of the complex (Figure 1). Each of the two samarium(III) ions occupies one of the TREN pockets and they exhibit an intermetallic distance of 5.1758(9) Å. The ligand framework is “twisted” along the metal–metal axis, which indicates a certain degree of flexibility. The distances of samarium to the amide nitrogen atoms are in the range of 2.244(5)–2.260(5) Å and those to the tertiary nitrogen atom are 2.514(4) Å. Each samarium center exhibits π-interaction with one of the bridging phenylenes with a short Sm1–C14 distance of 3.056(5) Å, which is well within the sum of the van der Waals radii of samarium and carbon of 4.67 Å.^21^ The solid-state structure also revealed vacant space in between the two samarium centers with available coordination sites along the metal–metal axes without any THF coordinated to the samarium ions, despite its use as a solvent in the preparation. This renders this compound an ideal platform to study the cooperative interaction with small molecules. As we observed the formation of the monometallated intermediate **2** in the synthesis of **1**, we were prompted to investigate the selective synthesis of the monosamarium derivative. Changing the stoichiometry to using equimolar amounts of H_6_L and Sm[N(SiMe_3_)_2_]_3_ under similar conditions yielded the monosamarium complex **2** selectively. Crystallization from hot benzene gave **2** as orange crystals in 76% isolated yield.

**Figure 2:**
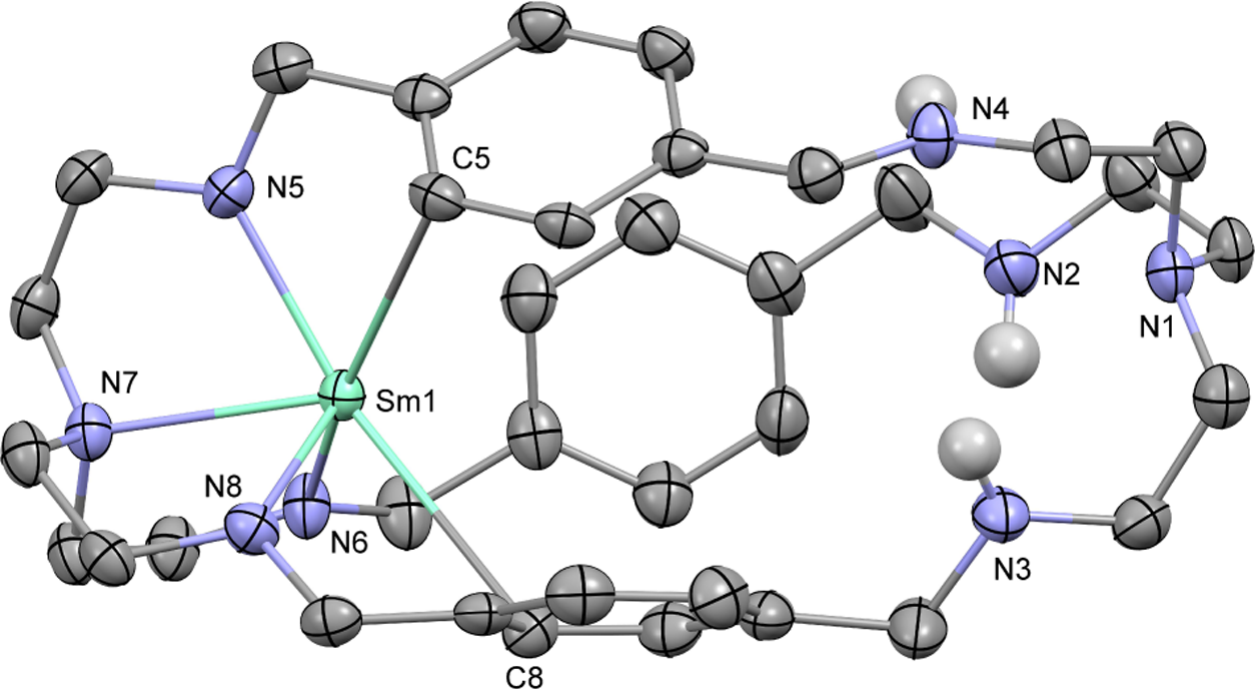
Molecular structure of **2**. Selected hydrogen atoms are omitted for clarity. Thermal ellipsoids drawn at 50% probability. Selected distances (Å) and angles (deg): Sm1–N5: 2.270(4), Sm1–N6: 2.280(3), Sm1–N8: 2.276(3), Sm1–N7: 2.521(4), Sm1–C5: 3.060(4), Sm1–C8: 3.070(5), N5–Sm1–N7: 69.4(1), N6–Sm1–N7: 69.7(1), and N7–Sm1–N8: 69.6(1).

This method also yielded crystals suitable for X-ray diffraction analysis and the molecular structure confirmed that only one of the TREN moieties was coordinated with a samarium ion (Figure 2). The distances of the samarium ion to the amide nitrogen atoms are in the range from 2.270(4) to 2.280(3) Å and the distance to the tertiary nitrogen atom is 2.521(4) Å, which is in both cases slightly longer than what was observed in **1**. With the second TREN pocket not being occupied, the one samarium center exhibits an interaction with two of the phenylenes with short Sm–C distances at 3.060(4) (C5) and 3.070(5) Å (C8). Interestingly, only two other monometallic complexes featuring this azacryptand ligand have been reported so far with cobalt.^22^ To assess the possibility of intermolecular metal ion exchange, we heated a solution of **2** in benzene to 70 °C for 1 day, but did not observe significant redistribution to **1** and H_6_L.

With the intermetallic space in the dinuclear complex **1** being available for coordination, we probed the cooperative ligand binding with cyanide, which is isoelectronic to carbon monoxide and dinitrogen. For this purpose, we treated **1** with equimolar amounts of potassium cyanide and 18-crown-6 at 70 °C. Heating was required due to the low solubility of potassium cyanide in THF even in the presence of crown ether and was applied until all of it was consumed, which was typically within 2 h. This yielded the cyanide adduct [LSm_2_-μ-η^1^:η^1^-CN][K(18-crown-6)(THF)_2_] (**3**) as yellow crystals in 86% after crystallization from THF/diethyl ether at −50 °C. The ion-separated compound is insoluble in aromatic and aliphatic solvents but readily dissolves in THF in contrast to its parent compound **1**.

**Figure 3:**
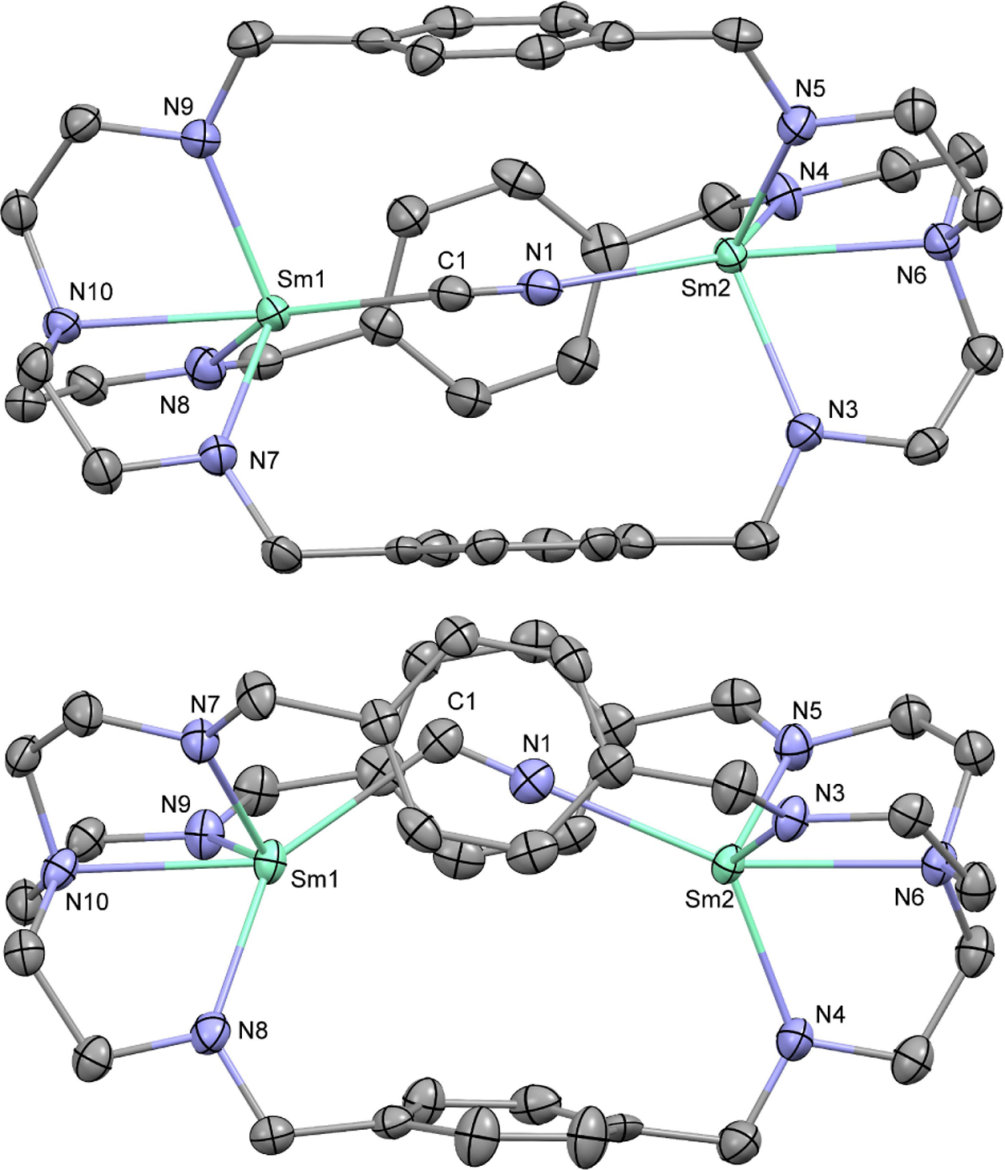
Molecular structure of an anionic substructure of **3** in side and top views. Hydrogen atoms and additional disordered cyanide are omitted for clarity. Thermal ellipsoids drawn at 50% probability. Selected distances (Å) and angles (deg): C1–N1: 1.20(2), Sm1–C1: 2.52(2), Sm2–N1: 2.56(1), Sm1–N7: 2.279(5), Sm1–N8: 2.312(6), Sm1–N9: 2.320(6), Sm1–N10: 2.526(4), Sm2–N3: 2.312(6), Sm2–N4: 2.306(5), Sm2–N5: 2.290(6), Sm2–N6: 2.533(4), Sm1···Sm2: 5.512(1), Sm1–C1–N1: 121(1), and Sm2–N1–C1: 175(1).

Crystals of the cyanide adduct **3** suitable for single-crystal X-ray diffraction were grown from a highly concentrated solution in THF at ambient temperature. The solid-state structure of **3** (Figure 3) shows the compound as a coordination polymer with the [LSm_2_-μ–η^1^:η^1^-CN]^−^ fragments being bridged with [K(18-crown-6)]^+^ groups and the asymmetric unit contains two sets of each fragment (see Figure S1). Within the rare-earth metal fragments, the bridging cyanide groups are not directly on the metal–metal axis but slightly “pushed out” of the center of the complexes and in between two of the bridging phenylenes, which are oriented almost co-planar. The ligand frameworks are stretched out with Sm···Sm distances extended from 5.1758(9) Å in **1** to 5.463(1)–5.512(1) Å in **3**. The cyanides are coordinated to both the rare-earth metal centers and are disordered. Previously reported multinuclear samarium(III) complexes featuring bridging cyanides exhibited nearly linear Sm–CN–Sm axes.^23−27^ Since the intermetallic distance is controlled by the ligand framework and is too short to support a linear arrangement, the cyanide appears in a range of orientations to adopt nearly linear coordination with either of the two metal centers. Modeling the disordered cyanides resulted in nearly linear arrangement of the cyanides with one of the two samarium atoms as depicted in Figure 3 with one of the two disordered cyanide groups. Refinement of these linear arrangements as N–C–Sm or C–N–Sm did not exhibit significant differences in the *R*-factor values, rendering them indistinguishable. DFT calculations by Yang and co-workers on fullerene-encapsulated yttrium and terbium cyanide compounds, in which the carbon and nitrogen atoms of the cyanides were also indistinguishable by X-ray diffraction analysis, showed the C–N–Ln arrangement in nearly linear coordination to be more stable than the N–C–Ln arrangement.^28^ Thus, we assigned carbon and nitrogen for near linear coordination to one samarium via the nitrogen atom (C1–N1–Sm2 in Figure 3) and the bent coordination (Sm1–C1–N1 in Figure 3) to the other samarium atom via the carbon atom. The bond distances between the cyanide nitrogen and the samarium atoms are in the range of 2.49(3)–2.56(1) Å, of which the N1–Sm2 bond depicted in Figure 3 represents the upper end, and the Sm–C_CN_ distances range from 2.47(7) to 2.52(2) Å, of which the Sm1–C1 bond depicted in Figure 3 exhibits the longest distance (see Figure S1 for the remaining Sm–N_CN_ and Sm–C_CN_ bond distances). These bond distances are similar to previously reported examples for crystallographically characterized compounds featuring cyanides bridging two samarium ions.^23−27^ The C–N bond distances in the cyanides are in the range of 1.17(7)–1.20(2) Å and a corresponding IR stretch vibration is observed at 2116 cm^–1^ (see Figure S18). In comparison to **1**, the distances of the samarium atoms to the amide nitrogen atoms are elongated and in the range of 2.281–2.325 Å and those to the tertiary nitrogen atoms in the range of 2.510(6)–2.533(4) Å.

### Spectroscopy

The ^1^H NMR spectroscopic analysis of **1** showed three broadened and paramagnetically shifted resonances at −10.23, −3.06, and 6.53 ppm, of which we assigned the two former to the methylene groups in the TREN moieties and the latter to the phenylene-bound methylene groups. The resonance of the bridging phenylenes is not observed. Even after prolonged measurement time, no ^13^C NMR data could be recorded for **1**. Using the Evans method, we calculated the effective magnetic moment, μ_eff_, of **1** to be 2.35 μ_B_.^29^ This is comparably low for a dinuclear compound considering that a typical range for samarium(III) compounds was reported at 1.4–1.7 μ_B_.^30^ The absence of the second paramagnetic samarium(III) center in **2** is reflected in a symmetry break and a declining paramagnetic influence toward the unoccupied ligand pocket. The strongly paramagnetically shifted resonances range from −8.00 to 11.37 ppm. Among the nine resonances, the ones at −8.00, −1.29, and 1.65 ppm, which we assigned to the methylene of the samarium-coordinated TREN moiety, are strongly broadened and partially exhibit too low integrals in comparison with the other signals. The effective magnetic moment of the mononuclear complex was calculated to be μ_eff_ = 1.80 μ_B_. This is considerably higher than expected, when comparing this with the dinuclear derivative **1** and might indicate magnetic interaction of the two metal centers in **1**. In case of **3**, the introduction of the cyanide group into **1** results in a more rigid structure, which is also reflected in the NMR data with less strongly broadened signals. The ^1^H NMR resonances observed for **3** are significantly less paramagnetically shifted when compared with the parent complex **1** with the four signals of the *ate*-complex covering a range from −4.91 to 5.97 ppm. Along with the broadened methylene resonances at −4.91, −0.14, and 5.13 ppm, the narrow resonance for the phenylene protons is now observed at 5.97 ppm. Considering that the solid-state structure would suggest symmetry breaks and, thus, many more ^1^H NMR signals than four for the *ate*-complex, it appears that the cyanide can readily move in between the two samarium atoms so that the phenylene bridges can easily move “over” the small coordinated anion at ambient temperature. The effective magnetic moment of complex **3** was calculated to be μ_eff_ = 2.00 μ_B_. This value is lower than that calculated for **1** and might indicate antiferromagnetic coupling resulting from cyanide-mediated superexchange between the two samarium(III) ions. This difference may also be reflected in the diminished paramagnetic shifting of the ^1^H NMR resonances in comparison with compound **1**. The crystalline material used for the NMR analysis was prepared by layering solutions of **3** in THF with diethyl ether, which is presumably the reason why the ^1^H and ^13^C NMR data shows 2 equiv of coordinated THF. Since the THF does not exhibit any paramagnetic shifting, it is most likely bound to the crown ether-embedded potassium ion forming the often observed [K(18-crown-6)(THF)_2_]^+^ moiety in which the coordinated potassium ion saturates the two apical positions with THF molecules.^31,32^

The UV–vis spectra (see Figures S11–S14) of the complexes are dominated by the absorptions of the bridging phenylenes in the range of ca. 245–280 nm. Here, **1** and **2** exhibit a very similar absorption behavior with three prominent maxima at 255, 261, and 273 nm for **1** and 257, 260, and 273 nm for **2**, whereas **3** shows narrower absorption bands with maxima at 247, 254, 261, 268, and 273 nm. This indicates that a broader distribution of different conformations with similar energies is present in solution at ambient temperature for complexes **1** and **2**, broadening the absorption bands. However, in the case of **3**, the distribution of different conformations is narrower by comparison, which suggests slightly higher energy differences for the accessible conformations of the complex. This appears feasible considering the effect of cyanide coordination to the arrangement of the ligand framework in the solid state. At higher wavelengths, **3** also exhibits weak peaks at 363 and 392 nm, which are not observed in the spectra for **1** and **2**.

### Electrochemical Analyses

**Figure 4:**
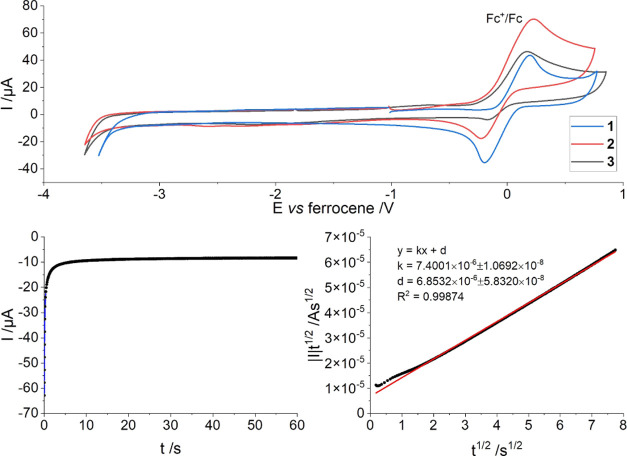
Top: Cyclic voltammetry of compounds **1**, **2**, and **3**. Data recorded from 2 mM solutions of both analyte and ferrocene in THF using ^*n*^Bu_4_NPF_6_ (100 mM) at scan rates of 50 (**2** and **3**) or 100 mVs^–1^ (**1**). Bottom left: Chronoamperometric data for **1** using a potential of −3.3 V vs Fc^+^/Fc for 60 s and the same analyte solution composition as for the cyclic voltammograms above. Bottom right: Plot of |*I*|*t*^1/2^ vs *t*^1/2^ of the data from the chronoamperometry of **1** fitted with a trend line (red).

Cyclic voltammetry experiments for the three samarium complexes did not exhibit any redox process related to the Sm^III^/Sm^II^ redox couple within the range of 0.8 to −3.5 V vs Fc^+^/Fc. The measurements were performed using ^*n*^Bu_4_NBPh_4_ or ^*n*^Bu_4_NPF_6_ (100 mM) as the supporting electrolyte in THF at ambient temperature with ferrocene in the same concentration as the analyte (2 mM) (Figures 4 and S20–S24). To exclude the possibility of a broadened redox band, a chronoamperometric measurement of a quiescent solution of **1** was performed over the period of 60 s at a potential of −3.3 V vs Fc^+^/Fc (Figure 4). This potential was chosen to avoid any reduction of the solvent or supporting electrolyte, so as to not confuse any such processes with the reduction of **1**. According to a published method,^33^ the data was plotted in the form of |*I*|*t*^1/2^ vs *t*^1/2^ from which the number of transferred electrons per analyte molecule (*n*) was estimated to 1.1 × 10^–2^ (Figure 4, see p15 in the ESI for the calculation details). The calculated value is significantly lower than that expected for the reduction of one samarium(III) atom (*n* = 1) or both (*n* = 2). Therefore, we conclude that no analyte reduction occurs at a potential of −3.3 V. This further supports the observations from the cyclic voltammetry that the Sm^III^/Sm^II^ redox couple is below the available measurement window in complex **1** and extends this conclusion to compounds **2** and **3**. For comparison, the Sm^III^/Sm^II^ redox couple for Sm[N(SiMe_3_)_2_]_3_ was reported for *E*_1/2_ = −2.1 V.^34^ However, in the case of the previously reported TREN-based europium complex N[(CH_2_)_2_NCH(C_6_H_2_-4,6-^*t*^Bu_2_-2-O)]_3_Eu, the Eu^III^/Eu^II^ redox couple, which is typically ca. 1 V higher than that of samarium, was observed only as a shoulder on the lower limit of the measurement window using similar conditions.^35^

## Conclusions

In summary, we presented the synthesis of a dinuclear azacryptand samarium complex **1** by the aminolysis reaction from Sm[N(SiMe_3_)_2_]_3_ and azacryptand H_6_L in a 2:1 molar ratio. Changing the ratio to 1:1 yielded the monometallic samarium complex **2**. The solid-state structure of disamarium complex **1** exhibits a vacant space in between the two metal centers along with the available coordination sites, despite the presence of otherwise strongly binding THF. With this ideal situation for cooperative small molecule activation, we investigated the incorporation of cyanide, which is isoelectronic to dinitrogen and carbon monoxide, forming metal-bridging adducts. We characterized the complexes by NMR, UV–vis, and IR spectroscopy as well as electrochemical analysis and single-crystal X-ray diffraction and determined the effective magnetic moments using the Evans method. These are the first examples of azacryptand-based f-metal complexes and we demonstrate their ability to bind cyanide in the presence of strongly coordinating solvents like THF. Moreover, the selective monometallation opens a pathway to mixed metal complexes combining different properties and reactivities. Consequently, these complexes represent a novel platform for the investigation of cooperative small molecule activation using f-elements and other early metals.

## Experimental Section

### General Details

All manipulations were carried out under an atmosphere of dry, oxygen-free nitrogen using standard Schlenk and glovebox techniques. Benzene-*d*_6_ was distilled from potassium, THF-*d*_8_ was dried with potassium, and pyridine-*d*_5_ degassed and dried over 4 Å molecular sieves. Dichloromethane and THF were purified by distillation from calcium hydride under nitrogen. All other solvents were purified by passing through columns of activated alumina.^36^ Other chemicals were obtained from different suppliers and used without further purification. N[(CH_2_)_2_NHCH_2_(*p*-C_6_H_4_)CH_2_NH(CH_2_)_2_]_3_N (H_6_L)^15^ and Sm[N(SiMe_3_)_2_]_3_^37^ were prepared according to published procedures. The NMR spectra were recorded on a Varian INOVA 500 or a Bruker AVANCE III 300 and were referenced to Me_4_Si (^1^H, ^13^C). The ^1^H NMR data required for the Evans method calculations of the magnetic moments, μ_eff_, were recorded using solutions of the analytes in pyridine-*d*_5_ with and without a sealed capillary containing pyridine-*d*_5_ to determine the shift differences between the solvent residue peaks.^29^ The average values of the shift differences of all three pyridine-*d* resonances were used for the calculations.^38,39^ For X-ray structure analyses, the crystals were mounted onto the tips of glass fibers. Data collection was performed with a Bruker-AXS SMART APEX CCD diffractometer using graphite-monochromated Mo Kα radiation (0.71073 Å). The data were reduced to F_o_^2^ and corrected for absorption effects with SAINT^40^ and SADABS,^41,42^ respectively. The structures were solved by direct methods and refined by the full-matrix least-squares method (SHELXL97 or SHELXL19).^43^ If not noted otherwise, all nonhydrogen atoms were refined with anisotropic displacement parameters. All hydrogen atoms were located in calculated positions to correspond to standard bond lengths and angles. Crystallographic data for the structures reported in this paper have been deposited with the Cambridge Crystallographic Data Center as supplementary publication no. CCDC 2109128 (**1**), 2109129 (**2**), and 2109130 (**3**). UV–vis spectra were recorded on an Agilent Cary 60 UV–vis spectrophotometer using THF to prepare analyte solutions for the measurements. Elementary analysis was carried out using a Heraeus VARIO ELEMENTAR. IR data was recorded on a Bruker Alpha-T FTIR spectrometer. The electrochemical analyses were performed using a BioLogic SP-150 potentiostat. The measurements were made on 2 mM solutions of the analyte in 10 mL of THF using *^n^*Bu_4_NBPh_4_ or *^n^*Bu_4_NPF_6_ (100 mM) as the supporting electrolyte in a glovebox. Here, a glassy carbon working electrode (*d* = 3 mm), a Pt-wire counter electrode, and a Ag-wire quasi-reference electrode were used and the data referenced against ferrocenium/ferrocene (Fc^+^/Fc = 0 V, 2 mM).

### Syntheses

#### Disamarium(III) Azacryptand Complex LSm_2_**1**

A vial equipped with a stirrer bar was charged with **H**_**6**_**L** (1.78 g, 2.97 mmol) and THF (5 mL) and a solution of Sm[N(SiMe_3_)_2_]_3_ (4.12 g, 6.53 mmol) in THF (5 mL) was added dropwise to the ligand solution. The yellow reaction mixture was filtered and heated to 70 °C for 5 h under vigorous stirring during which a bright yellow precipitate formed. After cooling to ambient temperature, the supernatant dark brown solution was decanted and the precipitate was washed four times with THF (5 mL) and one more time with Et_2_O (4 mL). The bright yellow solid was then dried under reduced pressure yielding 1.02 g (38%) of **1**. mp. 248 °C (dec.). ^1^H NMR (δ in ppm, benzene-*d*_6_, 298 K): −10.23 (br s, 12H, C*H*_2_), −3.06 (br s, 12H, C*H*_2_), 6.53 (br s, 12H, C*H*_2_). μ_eff_ = 2.35 μ_B_. UV–vis: λ_max,1_ = 249 nm (ε_1_ = 1.4 × 10^4^ L mol^–1^ cm^–1^, shoulder), λ_max,2_ = 255 nm (ε_2_ = 1.2 × 10^4^ L mol^–1^ cm^–1^), λ_max,3_ = 261 nm (ε_3_ = 1.1 × 10^4^ L mol^–1^ cm^–1^), λ_max,4_ = 264 nm (ε_4_ = 1.0 × 10^3^ L mol^–1^ cm^–1^, shoulder), λ_max,5_ = 273 nm (ε_5_ = 7.3 × 10^3^ L mol^–1^ cm^–1^, shoulder), λ_max,6_ = 306 nm (ε_6_ = 2.2 × 10^3^ L mol^–1^ cm^–1^, shoulder), λ_max,7_ = 338 nm (ε_7_ = 1.3 × 10^3^ L mol^–1^ cm^–1^, shoulder). IR (ATR, cm^–1^): 402, 422, 454, 503, 558, 625, 714, 742, 812, 842, 864, 904, 923, 964, 1017, 1044, 1047, 1098, 1127, 1146, 1184, 1224, 1254, 1289, 1317, 1341, 1436, 1454, 1501, 2119, 2340, 2687, 2742, 2789, 2808, 2876, 2939, 3013, 3275. Analysis calcd for C_36_H_48_N_8_Sm_2_ [893.56]: C 48.39, H 5.41, N 12.54. Found: C 48.73, H 5.25, N 12.53.

#### Samarium(III) Azacryptand Complex H_3_LSm **2**

A vial equipped with a stirrer bar was charged with **H**_**6**_**L** (598 mg, 1.00 mmol), Sm[N(SiMe_3_)_2_]_3_ (632 mg, 1.00 mmol), and THF (3 mL). The vial was sealed and then heated to 70 °C for 18 h. Then the dark brown mixture was allowed to cool to ambient temperature and orange crystals of **2** formed. The solution was then decanted and the orange crystals washed with diethyl ether yielding 500 mg (76%) of **2**. mp 232 °C (dec.). ^1^H NMR (δ in ppm, benzene-*d*_6_, 298 K): −8.00 (br, 6H, C*H*_2_), −1.89 (s, 3H, N*H*), −1.29 (br, 6H, C*H*_2_), 1.25 (s, 12H, C*H*_2_), 1.65 (br, 6H, C*H*_2_), 4.24 (d, *J* = 7 Hz, 6H, C*H*), 7.80 (s, 6H, C*H*_2_), 11.37 (d, *J* = 5 Hz, 6H, C*H*). ^13^C{^1^H} NMR (δ in ppm, benzene-*d*_6_, 298 K): 45.9, 50.5, 51.5, 66.1, 81.0, 124.4, 135.9, 145.3. μ_eff_ = 1.80 μ_B_. UV–vis: λ_max,1_ = 247 nm (ε_1_ = 1.4 × 10^4^ L mol^–1^ cm^–1^, shoulder), λ_max,2_ = 254 nm (ε_2_ = 1.2 × 10^4^ L mol^–1^ cm^–1^), λ_max,3_ = 260 nm (ε_3_ = 1.0 × 10^4^ L mol^–1^ cm^–1^), λ_max,4_ = 264 nm (ε_4_ = 9.8 × 10^3^ L mol^–1^ cm^–1^, shoulder), λ_max,5_ = 273 nm (ε_5_ = 7.0 × 10^3^ L mol^–1^ cm^–1^), λ_max,6_ = 298 nm (ε_6_ = 2.3 × 10^3^ L mol^–1^ cm^–1^). IR (ATR, cm^–1^): 390, 415, 489, 546, 570, 602, 619, 677, 721, 748, 784, 804, 845, 863, 911, 935, 973, 1019, 1054, 1069, 1098, 1125, 1138, 1197, 1211, 1223, 1258, 1281, 1292, 1325, 1363, 1384, 1418, 1439, 1452, 1509, 1611, 1648, 1802, 1902, 2080, 2695, 2731, 2799, 2875, 2946, 3015, 3093, 3281, 3610. Analysis calcd for C_36_H_51_N_8_Sm [746.22]: C 57.93, H 6.89, N 15.02. Found: C 58.88, H 6.71, N 14.63.

#### Disamarium(III) Azacryptand Complex Cyanide Adduct [LSm_2_CN][K(18-Crown-6)(THF)_2_] **3**

A vial equipped with a stirrer bar was charged with **1** (180 mg, 0.20 mmol), 18-crown-6 (53 mg, 0.20 mmol), potassium cyanide (13 mg, 0.20 mmol), and THF (4 mL). The reaction mixture was heated to 70 °C for 2 h during which the initial yellow suspension turned into a clear orange-brown solution. Then the solvent was evaporated under reduced pressure and the brown residue crystallized from THF and Et_2_O at −50 °C yielding yellow crystals of **3** (0.21 g, 86%). mp 102 °C (dec.). ^1^H NMR (δ in ppm, THF-*d*_8_, 298 K): −4.91 (br, 12H, C*H*_2_), −0.14 (br, 12H, C*H*_2_), 1.78 (m, 8H, THF), 3.61 (m, 8H, THF), 3.92 (s, 24H, 18-crown-6), 5.13 (br, 12H, C*H*), 5.97 (s, 12H, C*H*_2_). ^13^C{^1^H} NMR (δ in ppm, THF-*d*_8_, 298 K): 26.4, 46.0, 65.4, 68.3, 71.6, 73.9, 127.7, 144.1. μ_eff_ = 2.00 μ_B_. UV–vis: λ_max,1_ = 247 nm (ε_1_ = 1.4 × 10^4^ L mol^–1^ cm^–1^), λ_max,2_ = 254 nm (ε_2_ = 1.2 × 10^4^ L mol^–1^ cm^–1^), λ_max,3_ = 261 nm (ε_3_ = 1.1 × 10^4^ L mol^–1^ cm^–1^), λ_max,4_ = 268 nm (ε_4_ = 8.9 × 10^3^ L mol^–1^ cm^–1^), λ_max,5_ = 272 nm (ε_5_ = 7.1 × 10^3^ L mol^–1^ cm^–1^, shoulder), λ_max,6_ = 291 nm (ε_6_ = 2.8 × 10^3^ L mol^–1^ cm^–1^, shoulder), λ_max,7_ = 305 nm (ε_7_ = 2.0 × 10^3^ L mol^–1^ cm^–1^, shoulder), λ_max,8_ = 363 nm (ε_8_ = 9.1 × 10^2^ L mol^–1^ cm^–1^), λ_max,9_ = 392 nm (ε_9_ = 6.6 × 10^2^ L mol^–1^ cm^–1^). IR (ATR, cm^–1^): 377, 400, 496, 529, 560, 621, 750, 806, 843, 867, 907, 958, 1056, 1101, 1203, 1246, 1281, 1326, 1349, 1376, 1408, 1437, 1451, 1470, 1506, 2058, 2115, 2630, 2694, 2739, 2769, 2814, 2864, 2885, 2942, 3048. Analysis calcd for C_49_H_72_KN_9_O_6_Sm_2_ [1222.99]: C 48.12, H 5.93, N 10.31. Found: C 48.35, H 5.20, N 10.42.

#### N[(CH_2_)_2_NHCH_2_-*p*-C_6_H_4_CH_2_NH(CH_2_)_2_]_3_N H_6_L^15^

Additional analytical data: ^1^H NMR (δ in ppm, benzene-*d*_6_, 298 K): 1.71 (quint, 6H, ^3^*J*_HH_ = 8 Hz, N*H*), 2.43 (m, 12H, C*H*_2_), 2.65 (m, 12H, C*H*_2_), 3.67 (d, 12H, ^3^*J*_HH_ = 8 Hz, ArC*H*_2_) 7.07 (s, 12H, C*H*). ^1^H NMR (δ in ppm, chloroform-*d*, 298 K): 1.72 (br s, 6H, N*H*), 2.65 (m, 12H, C*H*_2_), 2.81 (m, 12H, C*H*_2_), 3.67 (s, 12H, ArC*H*_2_) 6.87 (s, 12H, C*H*). Couplings to amine protons were only observed for data recorded of solutions in benzene-*d*_6_. ^13^C{^1^H} NMR (δ in ppm, benzene-*d*_6_, 298 K): 48.2, 53.5, 54.7, 127.8, 139.4. ^13^C{^1^H} NMR (δ in ppm, chloroform-*d*, 298 K, APT): 48.0, 53.6, 54.3, 127.5, 139.6. UV–vis: λ_max,1_ = 257 nm (ε_1_ = 1.1 × 10^4^ L mol^–1^ cm^–1^), λ_max,2_ = 264 nm (ε_2_ = 1.1 × 10^4^ L mol^–1^ cm^–1^), λ_max,3_ = 267 nm (ε_3_ = 1.0 × 10^4^ L mol^–1^ cm^–1^, shoulder), λ_max,4_ = 273 nm (ε_4_ = 7.3 × 10^3^ L mol^–1^ cm^–1^), λ_max,5_ = 331 nm (ε_5_ = 6.0 × 10^2^ L mol^–1^cm^–1^). IR (ATR, cm^–1^): 393, 491, 592, 738, 7725, 799, 864, 884, 920, 974, 1019, 1054, 1098, 1127, 1160, 1224, 1291, 1328, 1359, 1387, 1431, 1450, 1515, 1671, 1910, 2084, 2654, 2734, 2798, 2880, 2943, 3010, 3228, 3271, 3283, 3306.

## Supporting Information Available

Additional electronic supporting information includes crystallographic data, NMR, UV–vis, and IR spectroscopic data as well as electrochemical analyses of the presented compounds (PDF)

## Accession Codes

CCDC 2109128–2109130 contain the supplementary crystallographic data for this paper. These data can be obtained free of charge via www.ccdc.cam.ac.uk/data_request/cif, or by emailing data_request@ccdc.cam.ac.uk, or by contacting The Cambridge Crystallographic Data Centre, 12 Union Road, Cambridge CB2 1EZ, UK; fax: +44 1223 336033.
