# Supplementary material for: Mono- and Disamarium Azacryptand Complexes: A Platform for Cooperative Rare-Earth Metal Chemistry
Source: Inorg Chem. 2022 Mar 28;61(14):5539–46. doi: 10.1021/acs.inorgchem.1c03989 (PMC9006214; doi:10.1021/acs.inorgchem.1c03989)
Supplement: Supplementary file 1 — ic1c03989_si_001.pdf [file ic1c03989_si_001.pdf]

Mono- and Disamarium Azacryptand Complexes: A Platform for Cooperative Rare-Earth Metal Chemistry

Johanna M. Uher, Matthias R. Steiner, and Johann A. Hlina*

Institute of Inorganic Chemistry, Graz University of Technology, Stremayrgasse 9, 8010 Graz, Austria.

Supporting Information

Table of Contents

Crystallography 2
NMR spectroscopy 4
UV-vis spectroscopy 9
IR spectroscopy 11
Electrochemical analyses 13
References 16

Crystallography:

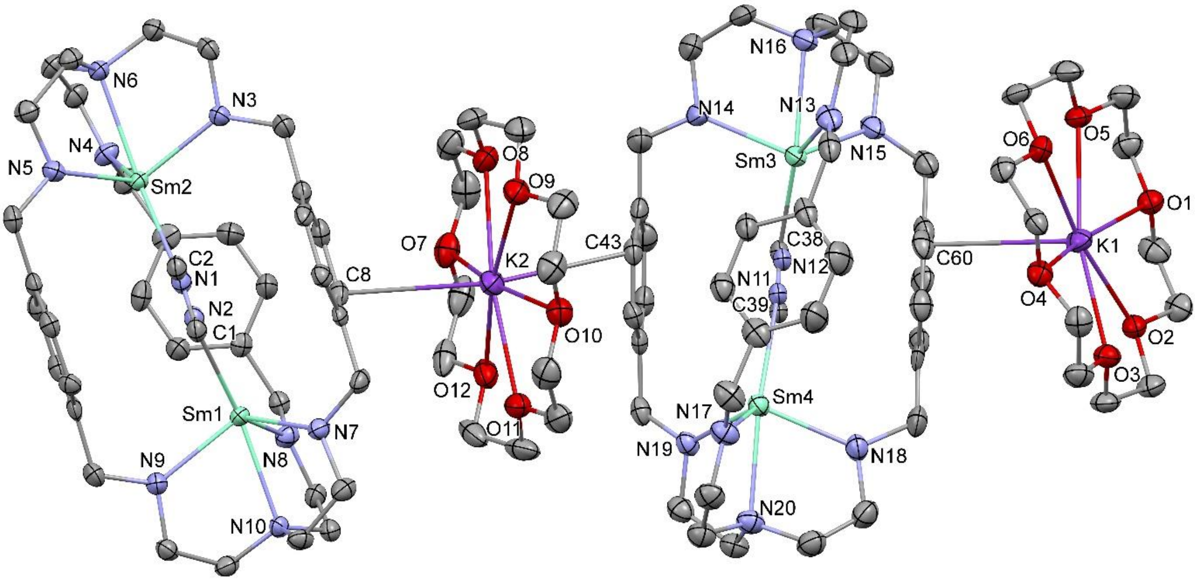

Figure S1. Molecular structure of **3**. Hydrogen atoms are omitted for clarity. Thermal ellipsoids drawn at 50 % probability. Selected distances (Å) and angles (deg): C1-N1: 1.20(2), C38-N11: 1.17(7), Sm1-C1: 2.52(2), Sm1-N2: 2.52(2), Sm2-N1: 2.56(1), Sm2-C2:2.49(3), Sm1-N7: 2.279(5), Sm1-N8: 2.312(6), Sm1-N9: 2.320(6), Sm1-N10: 2.526(4), Sm2-N3: 2.312(6), Sm2-N4: 2.306(5), Sm2-N5: 2.290(6), Sm2-N6: 2.533(4), Sm1-C1-N1: 121(1), Sm2-N1-C1: 175(1), Sm3-C38: 2.47(7), Sm3-N12: 2.50(2), Sm4-N11: 2.72(4), Sm4-C39: 2.50(3) Sm3-N13: 2.316(6), Sm3-N14: 2.325(5), Sm3-N15: 2.274(6), Sm3-N16: 2.510(5), Sm4-N17: 2.308(6), Sm4-N18: 2.317(5), Sm4-N19: 2.296(6), Sm4-N20: 2.527(5), Sm3-C38-N11: 109(4), Sm4-N11-C38: 167(4).

Table S1. Crystallographic data of **1**, **2**, and **3**.

	**1**	**2**	**3**
CCDC number	2109128	2109129	2109130
Empirical formula	$C_{36}H_{48}N_8Sm_2$	$C_{36}H_{51}N_8Sm$	$C_{98}H_{144}K_2N_{18}O_{12}Sm_4$
Formula weight	893.52	746.20	2445.90
Temperature /K	150(2)	200(2)	149(2)
Crystal system	monoclinic	monoclinic	monoclinic
Space group	*I*2/*a*	*C*2/*c*	$P2_1/n$
a /Å	17.522(4)	34.654(7)	25.427(5)
b /Å	9.4586(18)	9.9071(18)	18.680(4)
c /Å	21.097(4)	25.151(6)	27.219(6)
α /°	90	90	90
β /°	92.006(6)	120.862(6)	113.369(3)
γ /°	90	90	90
Volume /Å^3	3494.3(12)	7412(3)	11868(4)
Z	4	8	4
ρ_{calc}g /cm^3	1.698	1.337	1.369

M /mm^{-1}	3.366	1.619	2.078
F(000)	1776	3080	4960
Crystal size /mm^3	0.46 x 0.14 x 0.10	0.41 x 0.18 x 0.16	0.22 x 0.20 x 0.16
2Θ range for data collection /°	1.93 to 26.35	2.22 to 26.37	1.40 to 26.38
Index ranges	-21<=h<=21, -11<=k<=11, -26<=l<=26	-40<=h<=43, -12<=k<=12, -31<=l<=31	-31<=h<=31, -23<=k<=23, -34<=l<=33
Reflections collected	13521	28794	93155
Independent reflections	3561 [R(int) = 0.0366]	7537 [R(int) = 0.0257]	24209 [R(int) = 0.0450]
Data/restraints/parameters	3561 / 0 / 208	7537 / 0 / 406	24209 / 64 / 1213
Goodness-of-fit on F^2	1.259	1.077	1.134
Final R indexes [I>=2σ (I)]	R1 = 0.0414, wR2 = 0.0857	R1 = 0.0368, wR2 = 0.0924	R1 = 0.0557, wR2 = 0.1208
Final R indexes [all data]	R1 = 0.0429, wR2 = 0.0863	R1 = 0.0400, wR2 = 0.0946	R1 = 0.0661, wR2 = 0.1250
Largest diff. peak/hole / e Å^{-3}	1.384 and -0.957	0.901 and -0.637	1.80 and -0.90

NMR spectroscopy:

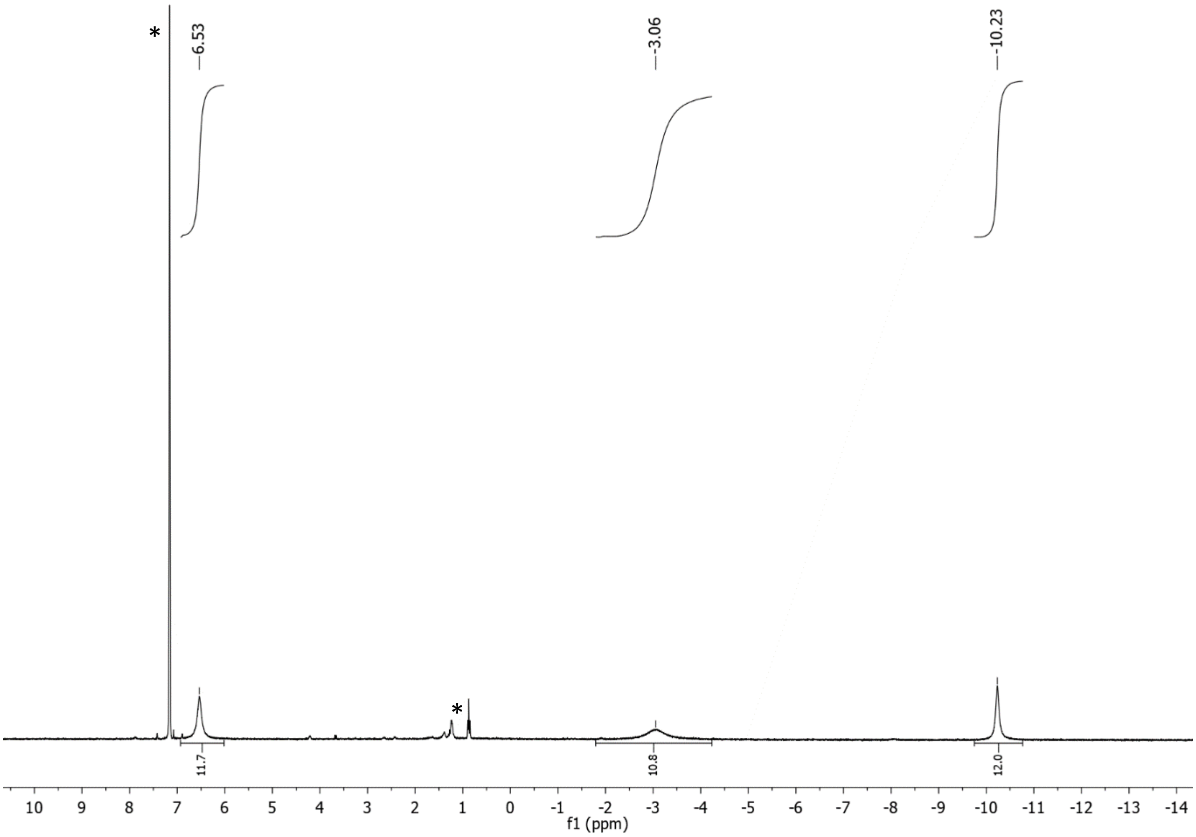

Figure S2. ^{1}H NMR spectrum of **1** in C_6D_6 recorded at 298 K. NMR solvent and impurities are marked with an asterisk.

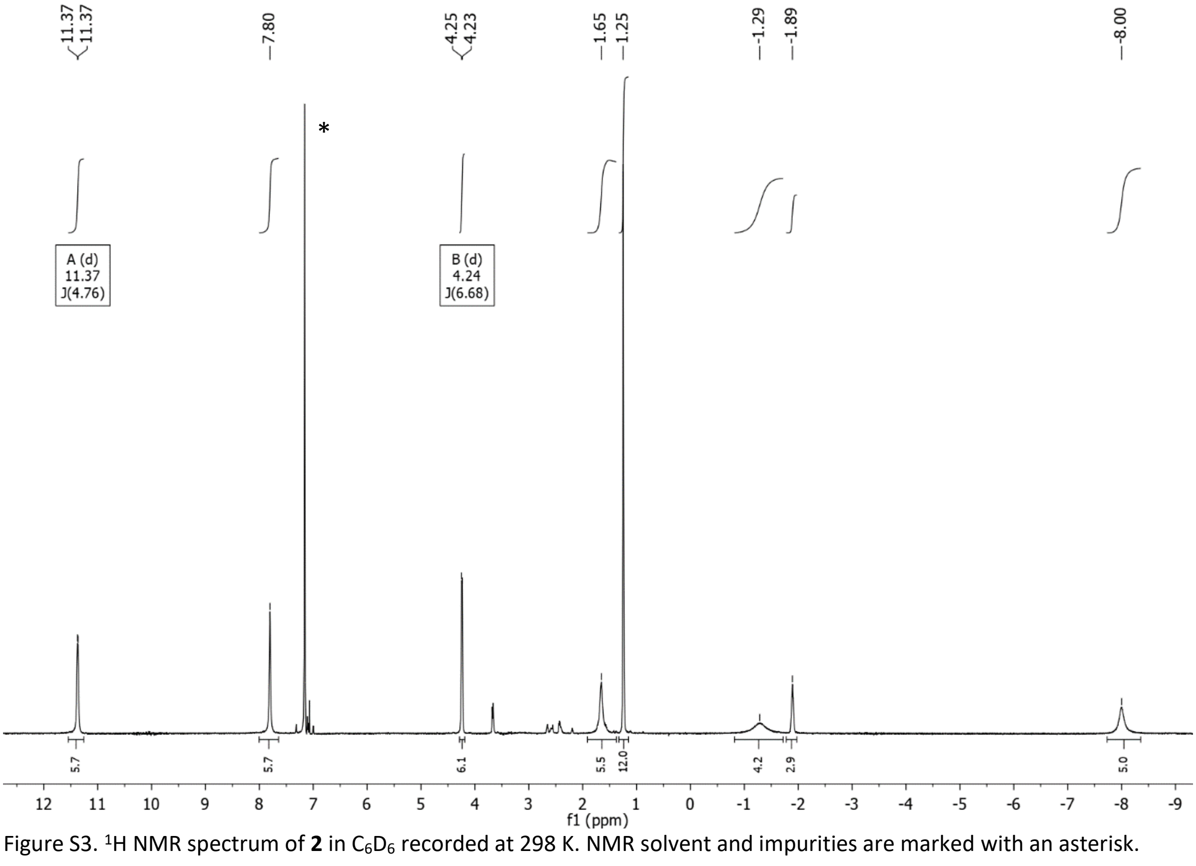

Figure S3. ^{1}H NMR spectrum of **2** in C_6D_6 recorded at 298 K. NMR solvent and impurities are marked with an asterisk.

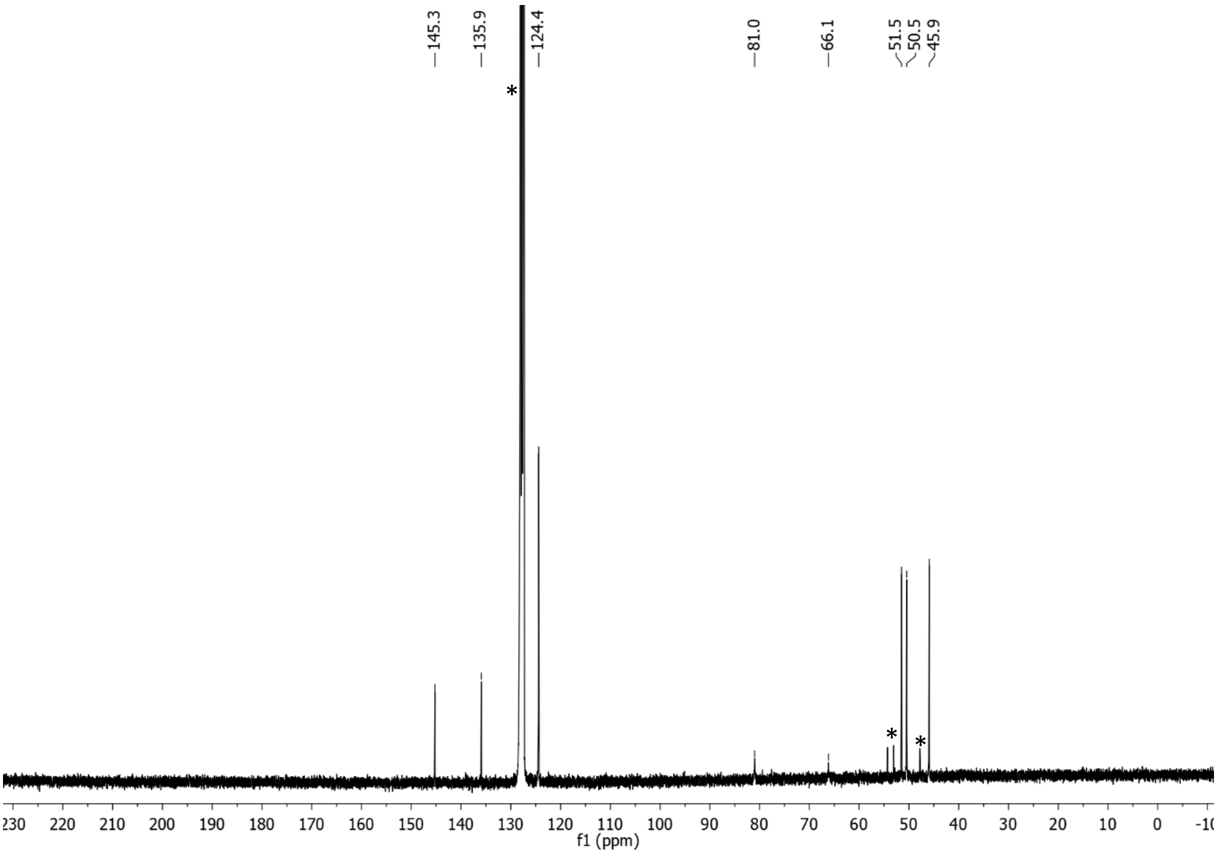

Figure S4. ^{13}C NMR spectrum of **2** in C_6D_6 recorded at 298 K. NMR solvent and impurities are marked with an asterisk.

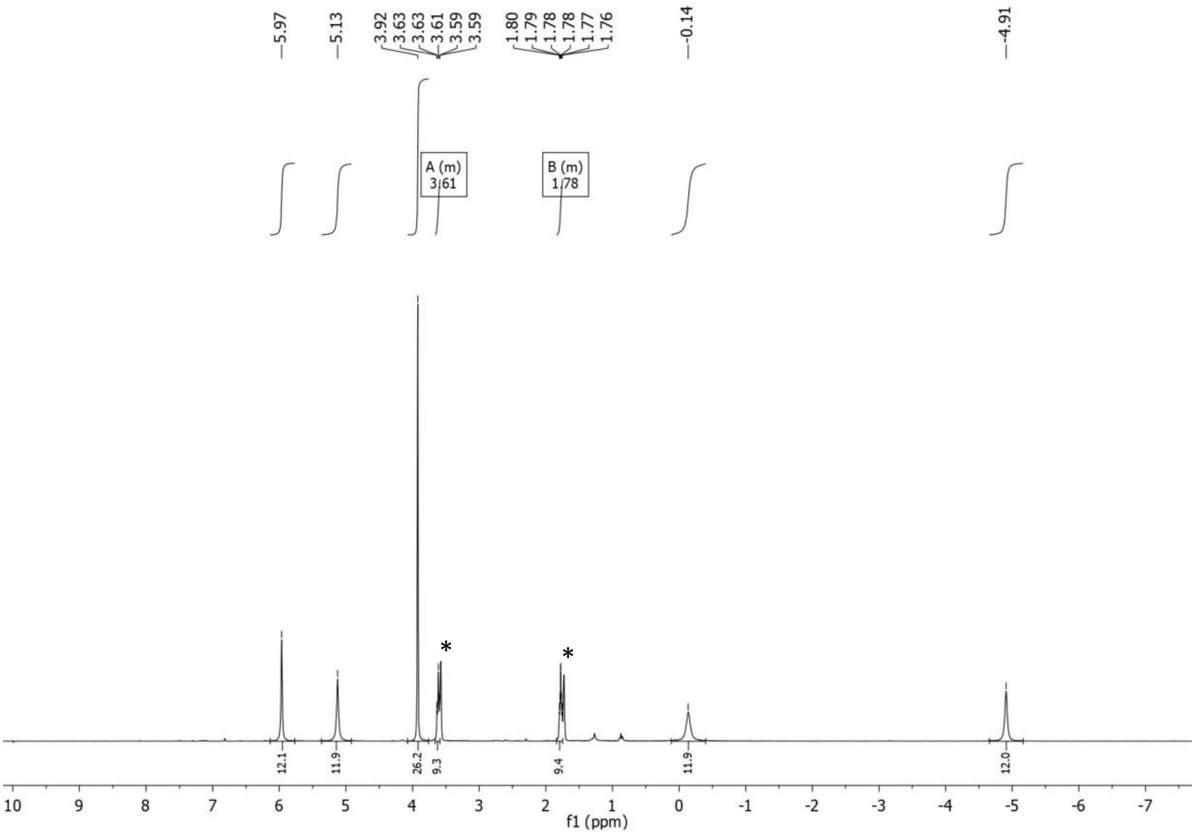

Figure S5. ^{1}H NMR spectrum of **3** in THF-*d8* recorded at 298 K. NMR solvent is marked with an asterisk.

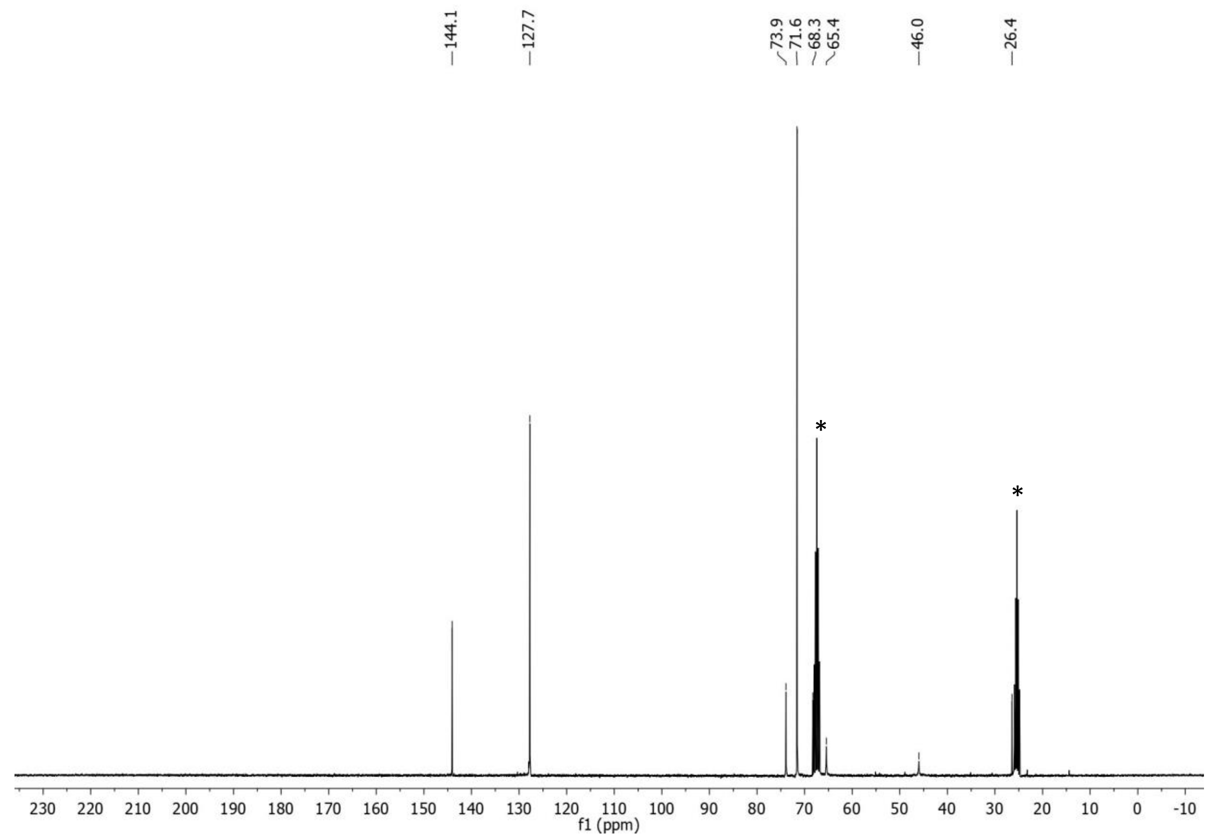

Figure S6. ^{13}C NMR spectrum of **3** in THF-*d8* recorded at 298 K. NMR solvent is marked with an asterisk.

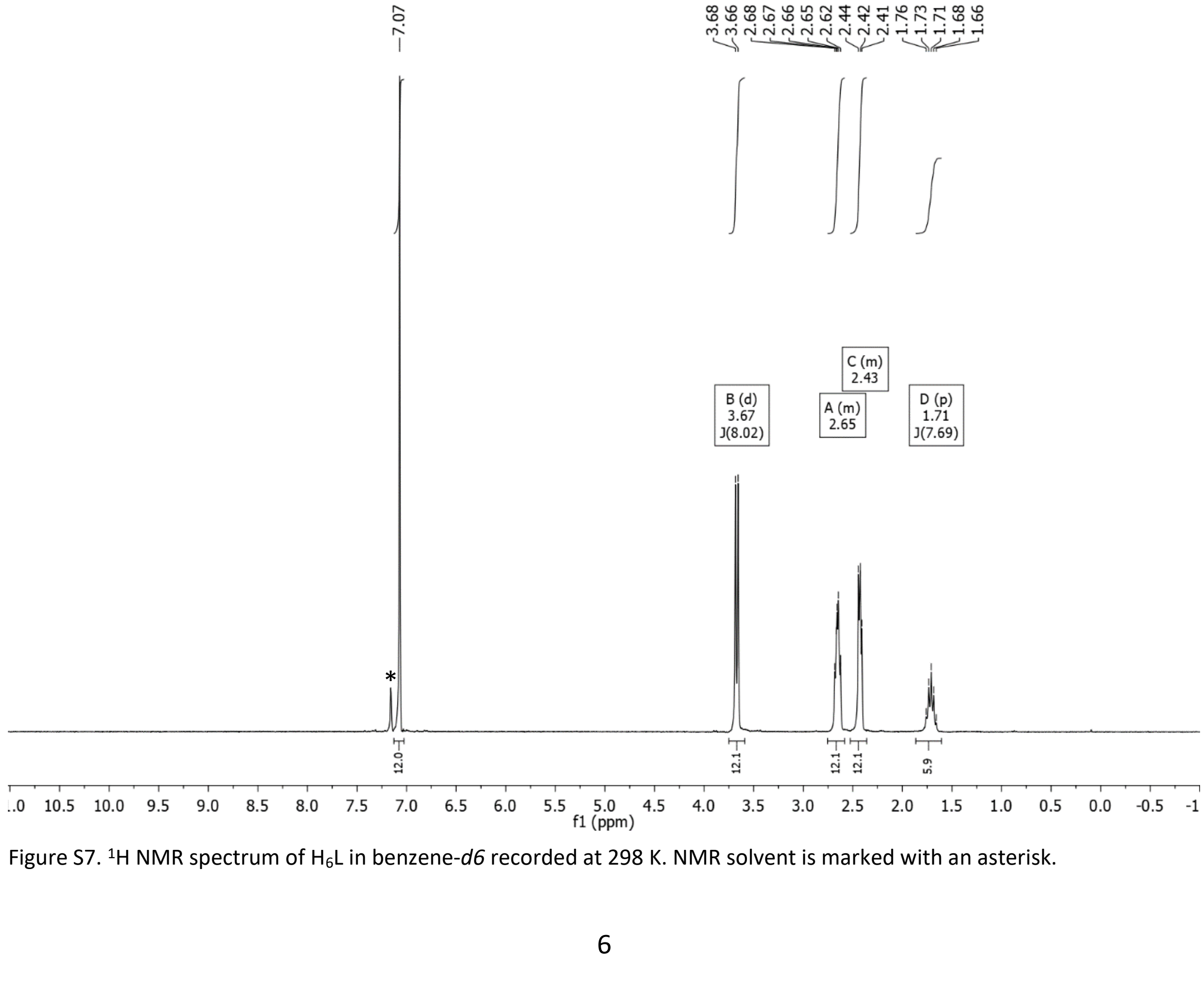

Figure S7. ^{1}H NMR spectrum of H_6L in benzene-*d6* recorded at 298 K. NMR solvent is marked with an asterisk.

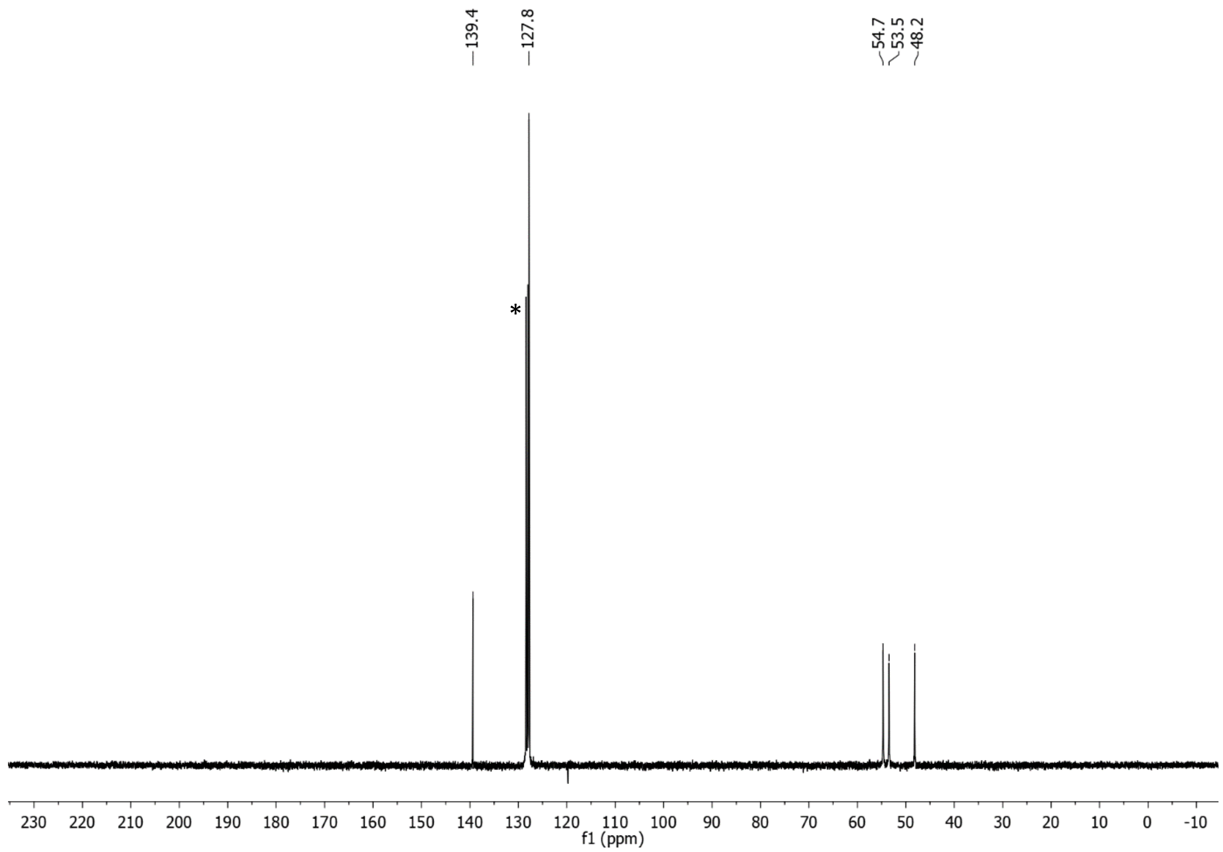

Figure S8. ^{13}C NMR spectrum of H_6L in benzene-*d6* recorded at 298 K. NMR solvent is marked with an asterisk.

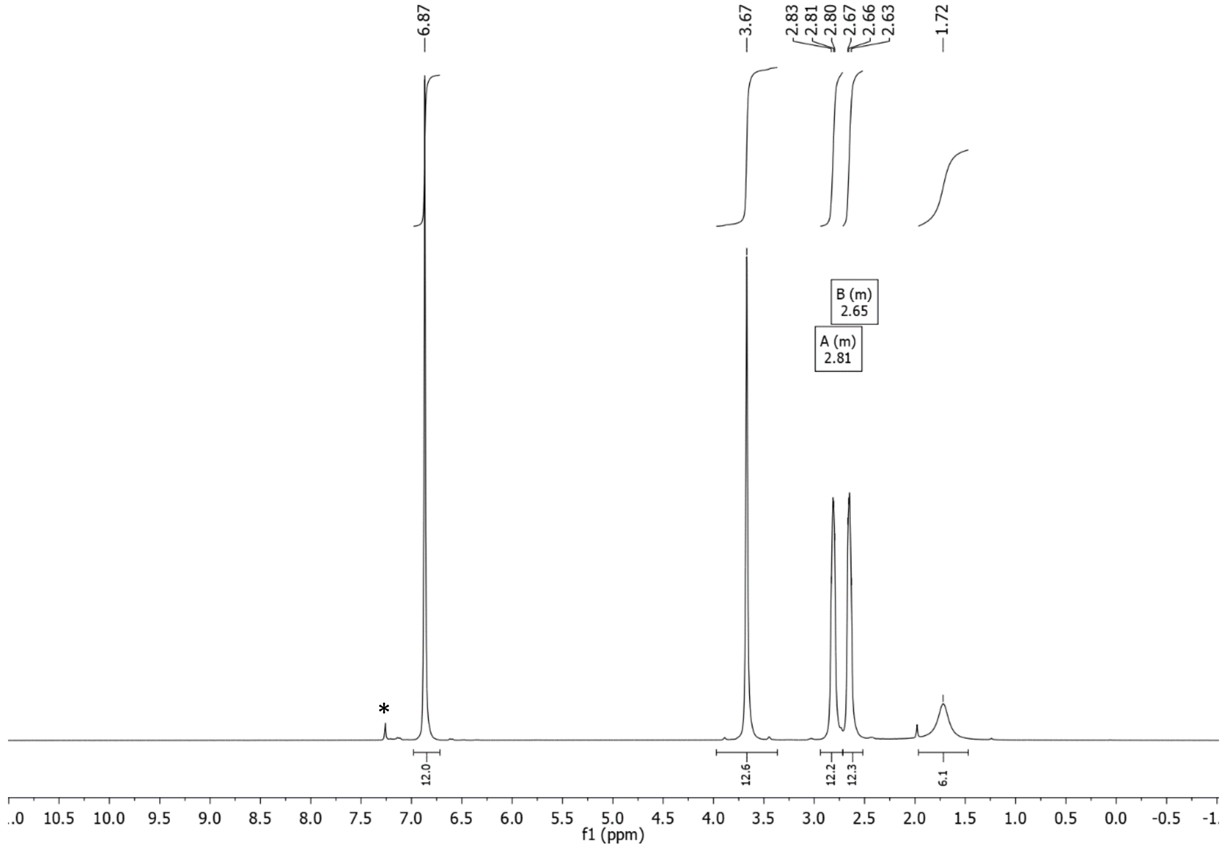

Figure S9. ^{1}H NMR spectrum of H_6L in chloroform-*d* recorded at 298 K. NMR solvent is marked with an asterisk.

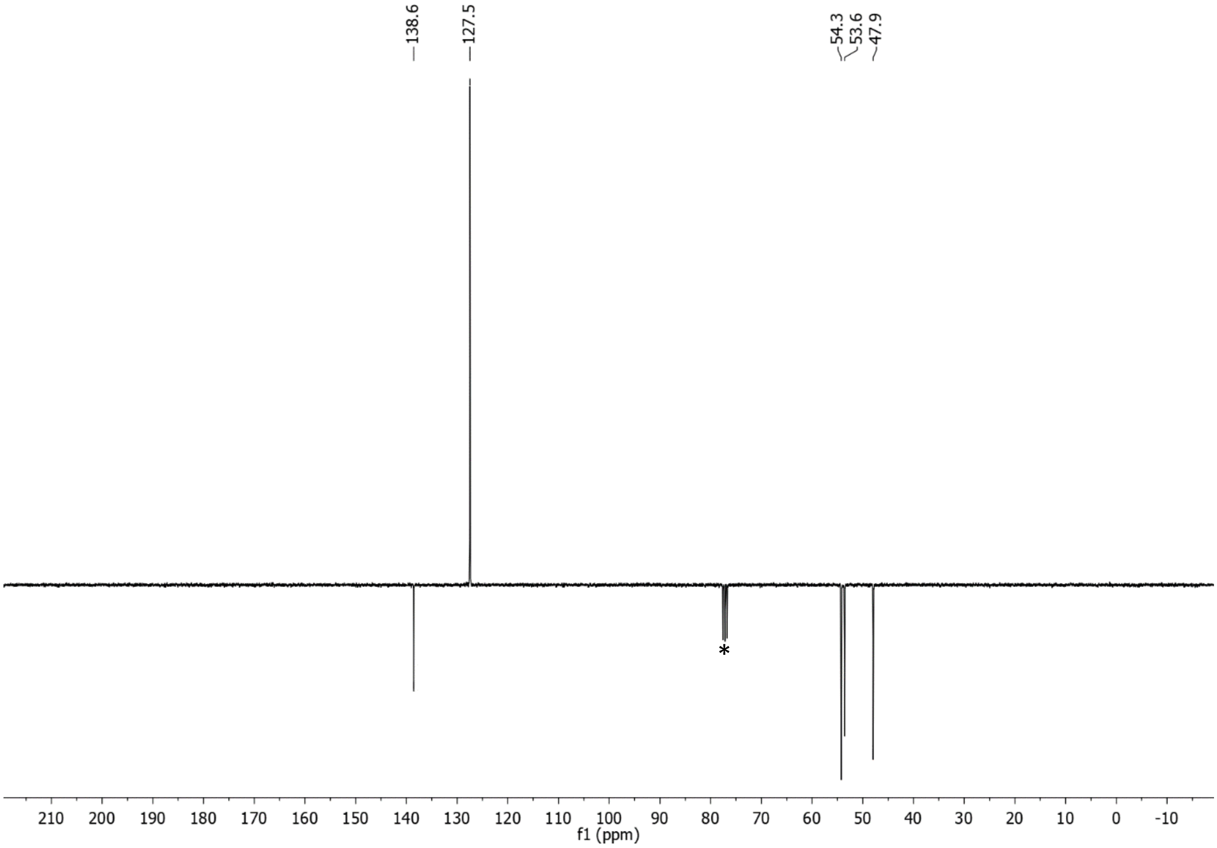

Figure S10. ^{13}C APT NMR spectrum of H_6L in chloroform-*d* recorded at 298 K. NMR solvent is marked with an asterisk.

UV-vis spectroscopy:

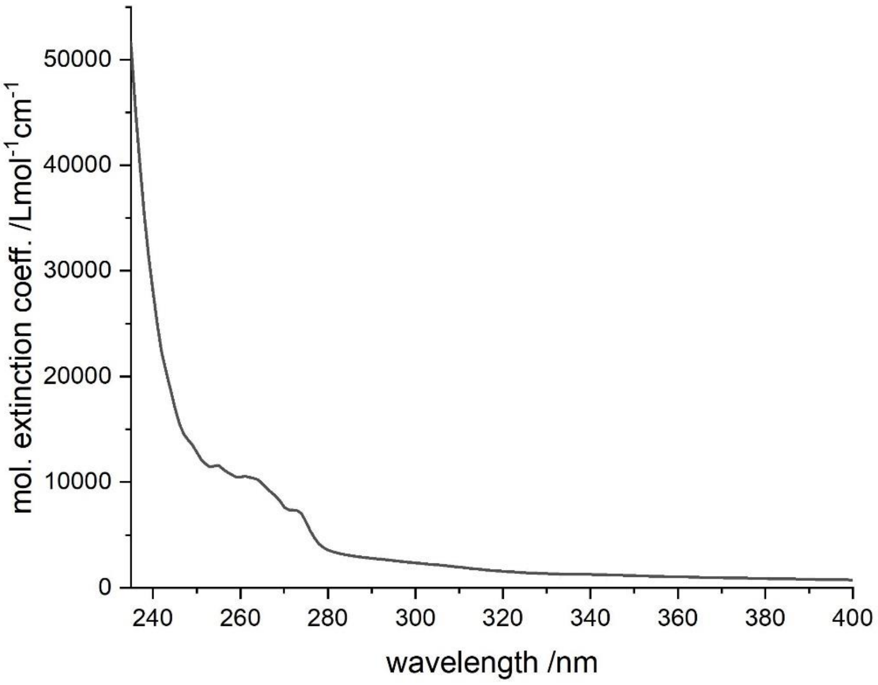

Figure S11. UV-vis spectrum of the compound **1** recorded from a 0.02 mM solution in THF at ambient temperature.

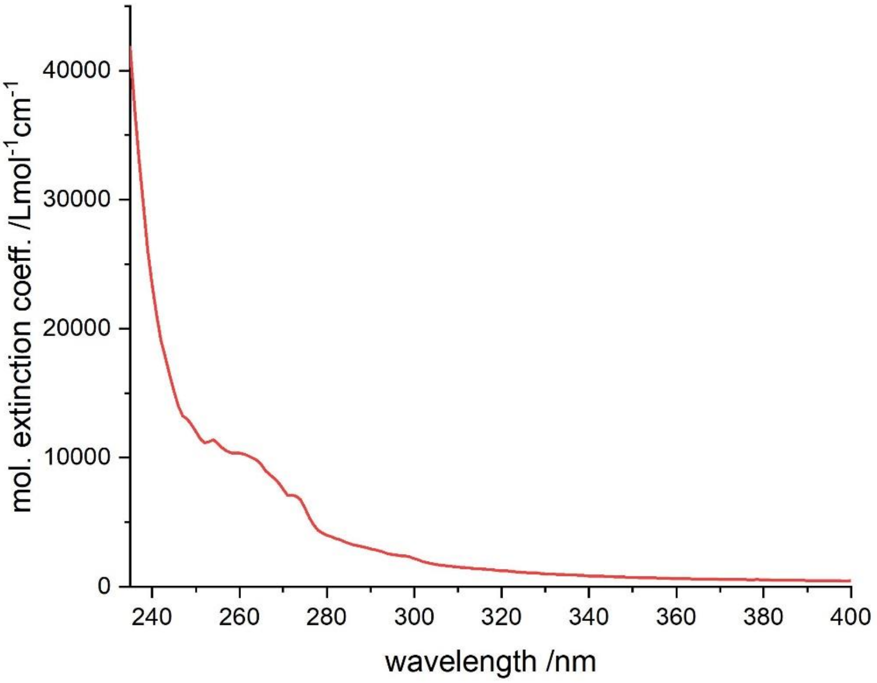

Figure S12. UV-vis spectrum of the compound **2** recorded from a 0.03 mM solution in THF at ambient temperature.

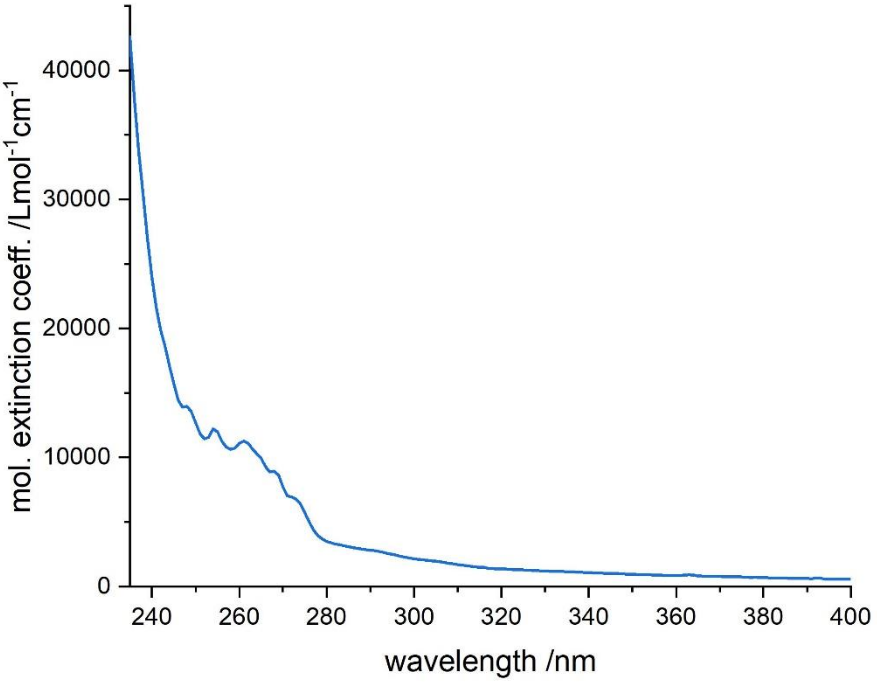

Figure S13. UV-vis spectrum of the compound **3** recorded from a 0.03 mM solution in THF at ambient temperature.

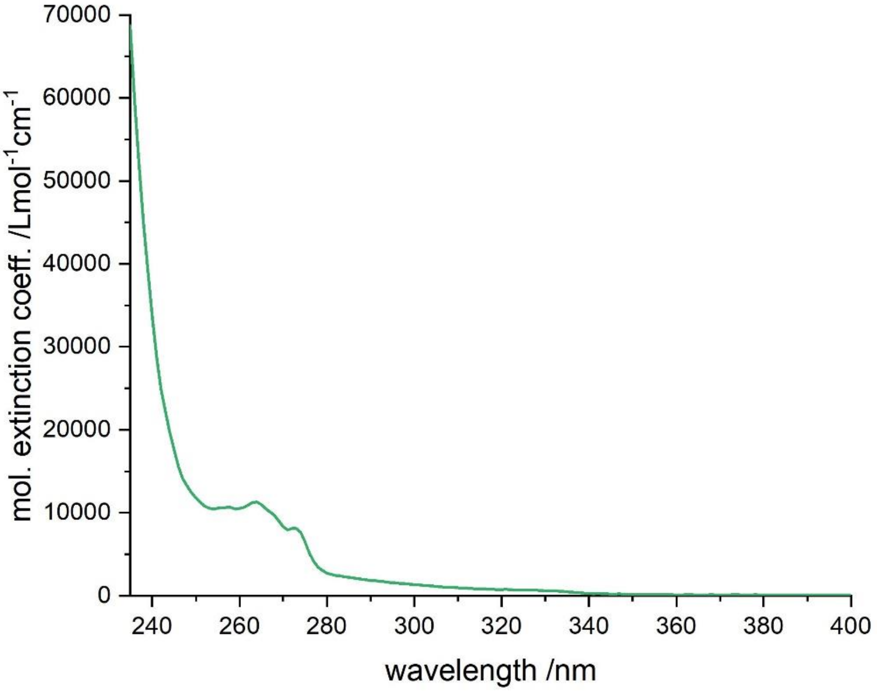

Figure S14. UV-vis spectrum of the compound H_6L recorded from a 0.05 mM solution in THF at ambient temperature.

IR spectroscopy:

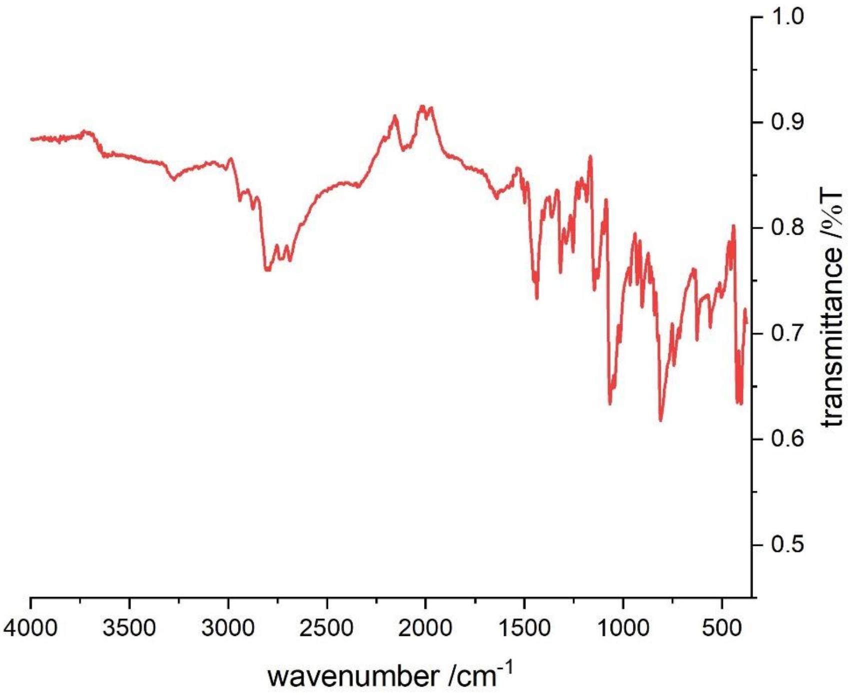

Figure S15. IR spectrum of **1** recorded neat from crystalline material using ATR.

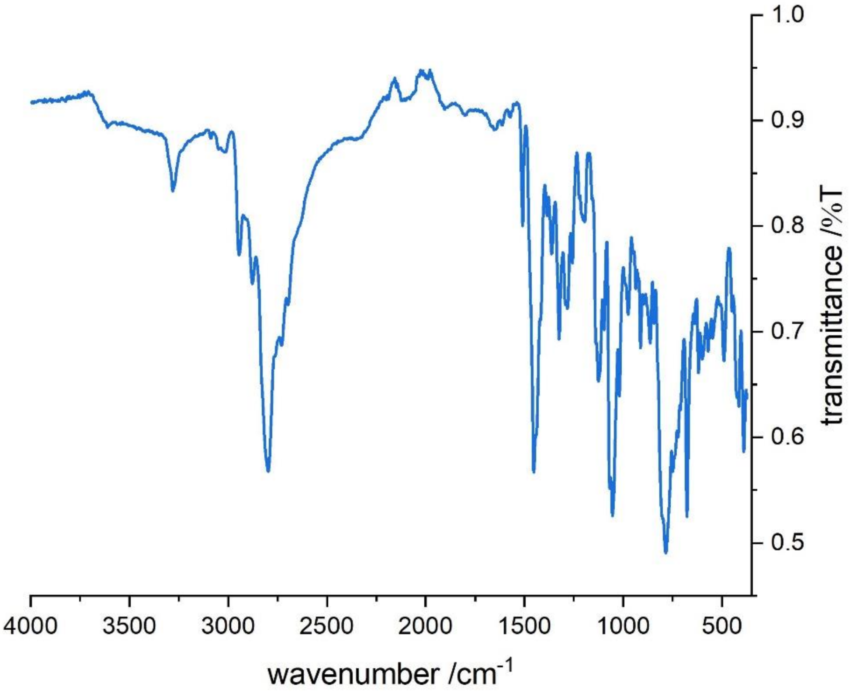

Figure S16. IR spectrum of **2** recorded neat from crystalline material using ATR.

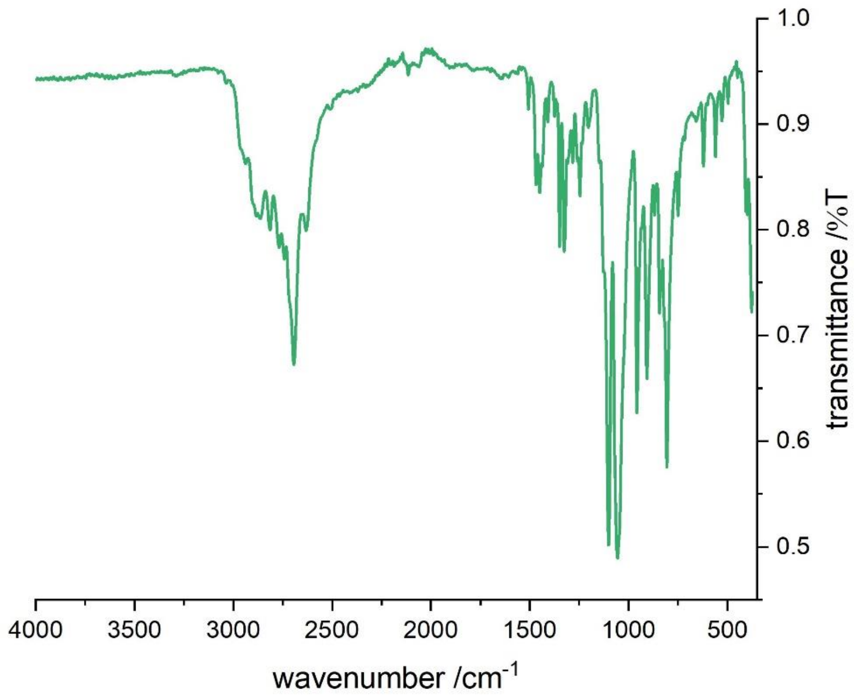

Figure S17. IR spectrum of **3** recorded neat from crystalline material using ATR.

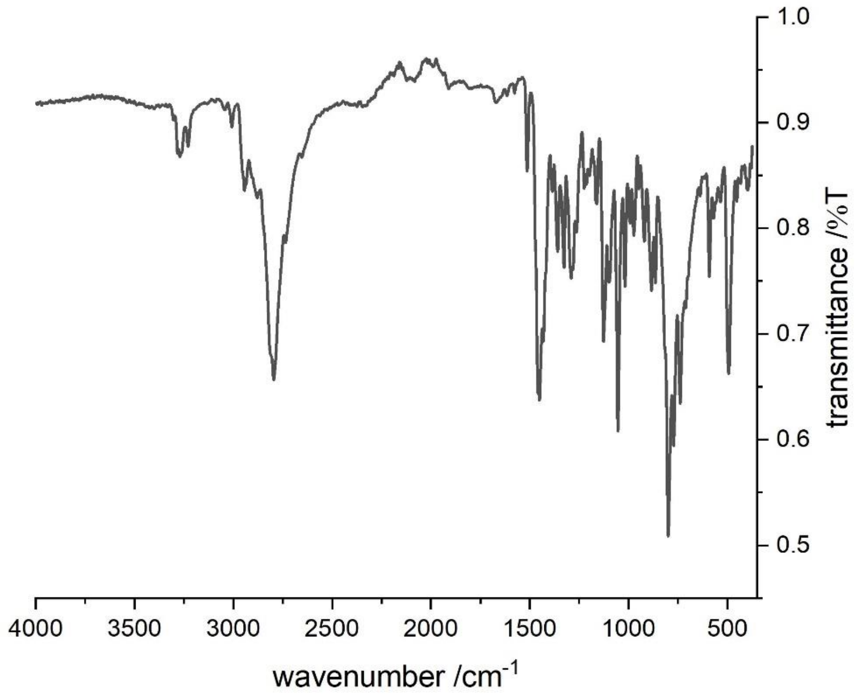

Figure S18. IR spectrum of H_6L recorded neat from crystalline material using ATR.

Electrochemical analyses:

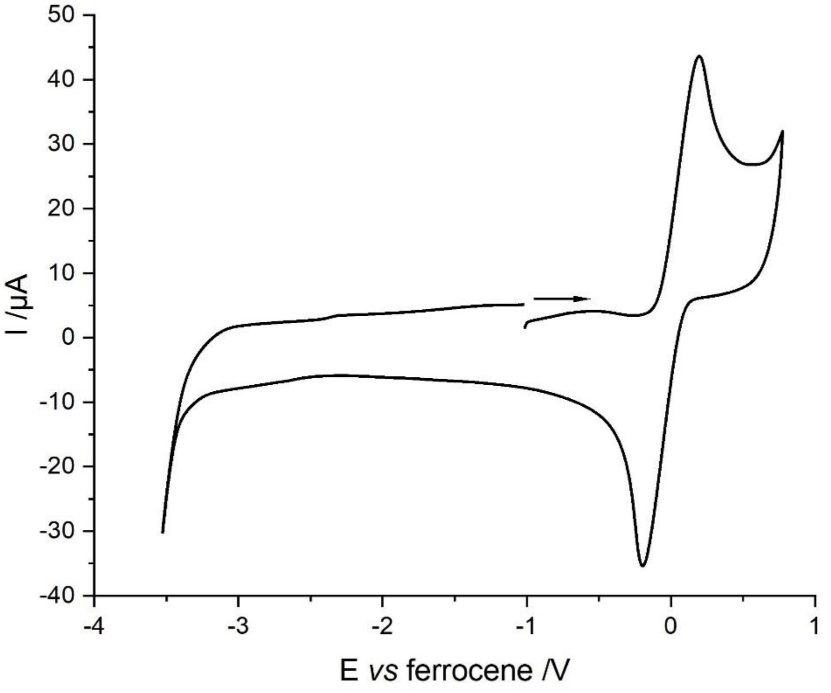

Figure S19. Cyclic voltammogram for **1** in the presence of ferrocene measured at a scan rate of 100 mVs^{-1}. The arrow indicates the scan direction.

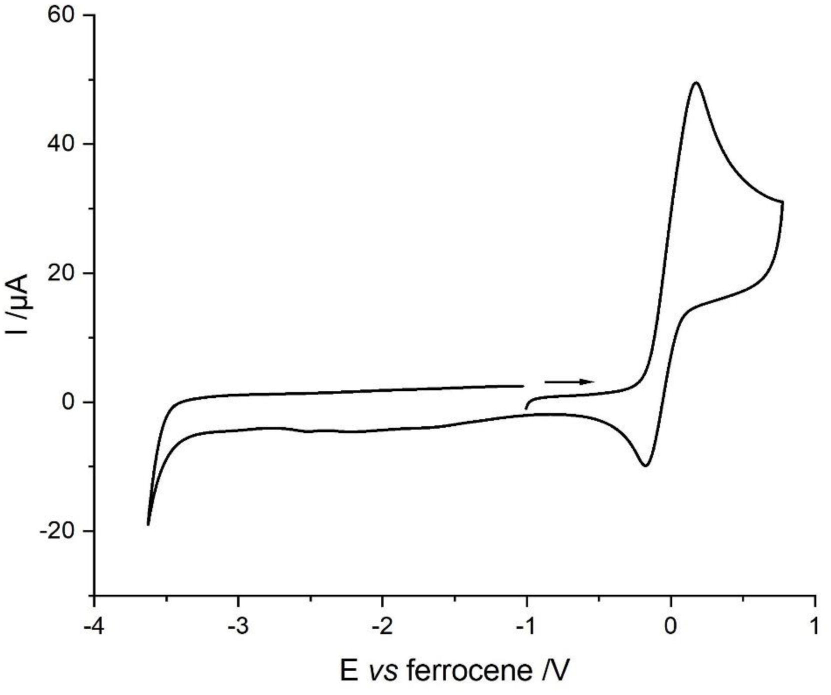

Figure S20. Cyclic voltammogram for **2** in the presence of ferrocene measured at a scan rate of 50 mVs^{-1}. The arrow indicates the scan direction.

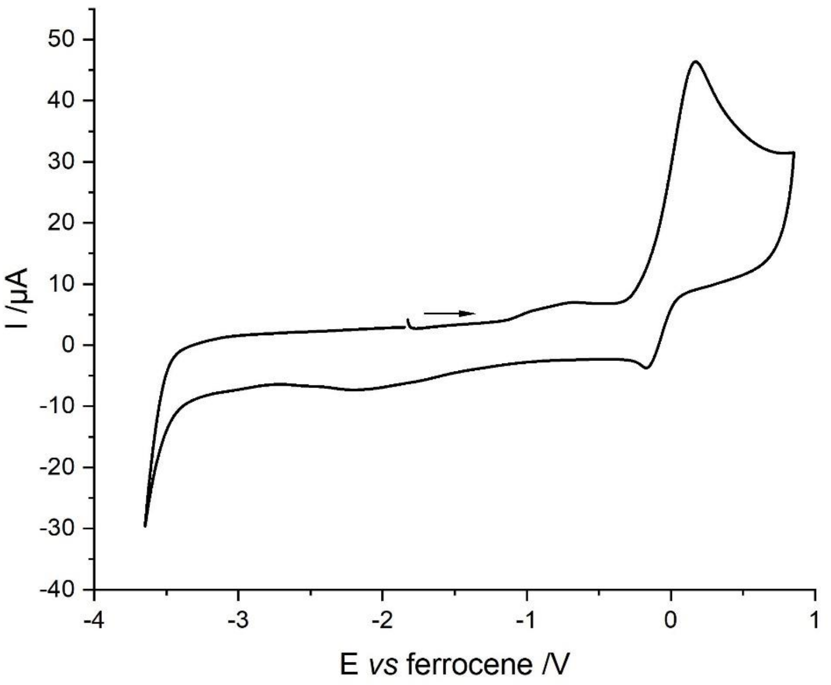

Figure S21. Cyclic voltammogram for **3** in the presence of ferrocene measured at a scan rate of 50 mVs^{-1}. The arrow indicates the scan direction.

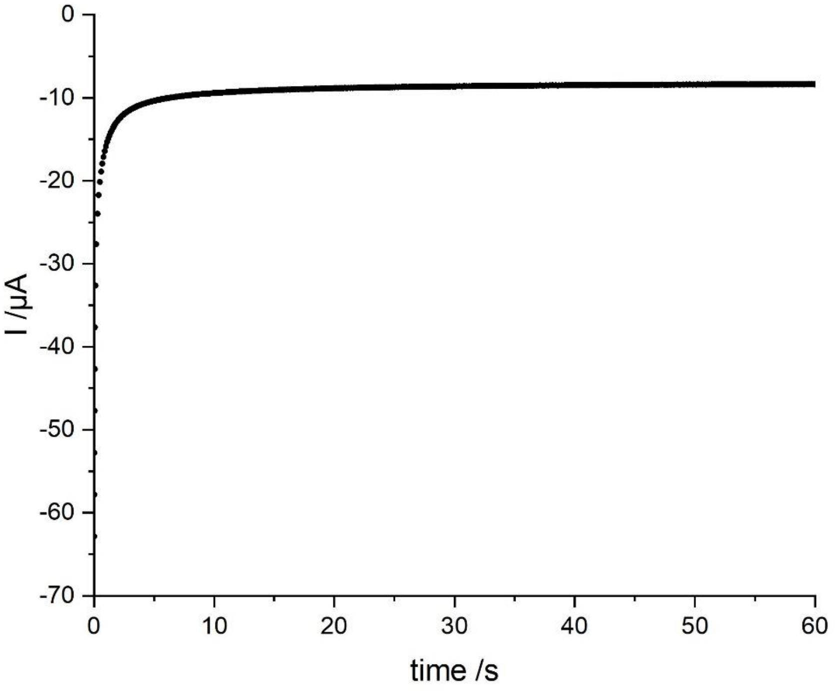

Figure S22. Chronoamperometric data for **1** at a potential of -3.3 V *vs* Fc^+/Fc recorded for 60 s.

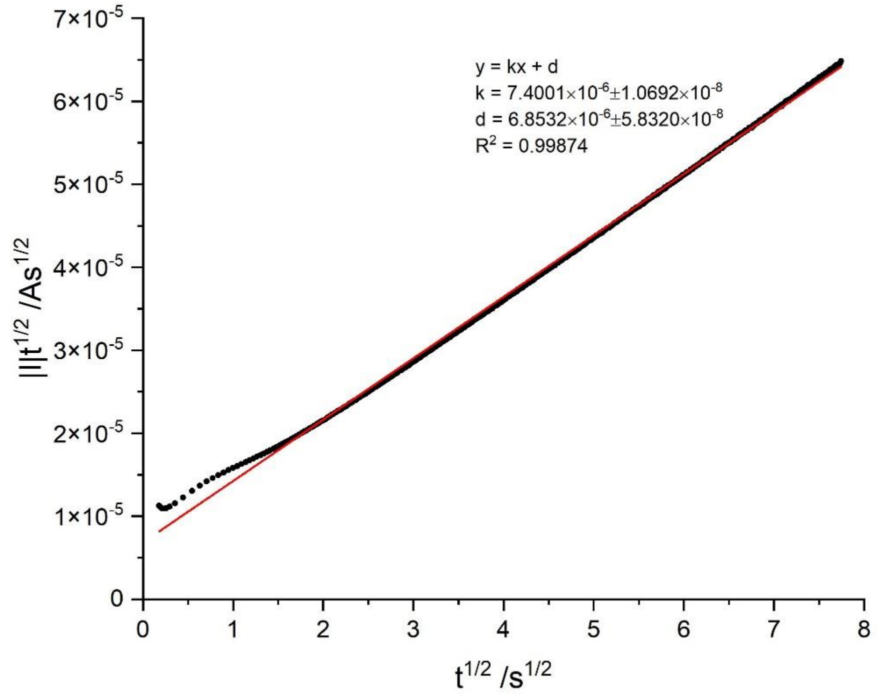

Figure S23. Plot of $t^{1/2}$ *vs* $|I|t^{1/2}$ derived from the chronoamperometric data recorded for **1**.

From the data from a chronoamperometric measurement using a planar disk electrode the number of electrons transferred per molecule may be calculated by

$$|I|t^{1/2} = \frac{nFAD^{1/2}C}{\pi^{1/2}}\left[1 + b\frac{(Dt)^{1/2}}{R}\right]$$

where *I* is the current, *t* is the time, *n* is the number of transferred electrons per molecule of analyte, *F* is the Faraday constant, *A* is the area of the disc electrode (0.071 cm^2), *D* is the diffusion coefficient, *C* is the initial analyte concentration (2.0 mM), *b* is an empirical constant, and *R* is the radius of the disc electrode (0.15 cm).[8] Constant *b* was reported to typically be a value of approximately 2, which was also used for this calculation. Transformation of the equation into the form of a linear equation for use of the data from the trend line in Figure S23 for *n* gives

$$n = \frac{bd^{1/2}}{kFR^3C\pi^{1/2}}$$

where *k* is the slope of the trend line ($7.40 \cdot 10^{-6}$ $s^{1/2}$) and *d* is its intercept ($6.85 \cdot 10^{-6}$ $As^{1/2}$). Using these data, *n* was calculated to a value of $1.1 \cdot 10^{-2}$.

References:

(1) Pangborn, A. B.; Giardello, M. A.; Grubbs, R. H.; Rosen, R. K.; Timmers, F. J. Safe and Convenient Procedure for Solvent Purification. *Organometallics* **1996**, *15* (5), 1518–1520. https://doi.org/10.1021/om9503712.

(2) Jazwinski, J.; Lehn, J.-M.; Lilienbaum, D.; Ziessel, R.; Guilhem, J.; Pascard, C. Polyaza Macrobicyclic Cryptands: Synthesis, Crystal Structures of a Cyclophane Type Macrobicyclic Cryptand and of Its Dinuclear Copper(I) Cryptate, and Anion Binding Features. *J. Chem. Soc., Chem. Commun.* **1987**, No. 22, 1691–1694. https://doi.org/10.1039/c39870001691.

(3) Schuetz, S. A.; Day, V. W.; Sommer, R. D.; Rheingold, A. L.; Belot, J. A. Anhydrous Lanthanide Schiff Base Complexes and Their Preparation Using Lanthanide Triflate Derived Amides. *Inorg. Chem.* **2001**, *40* (20), 5292–5295. https://doi.org/10.1021/ic010060l.

(4) *SAINTPLUS: Software Reference Manual, Version 6.45, Bruker-AXS, 1997-2003*; Madison, WI.

(5) Blessing, R. H. An Empirical Correction for Absorption Anisotropy. *Acta Crystallogr., Sect. A: Found. Crystallogr.* **1995**, *51* (1), 33–38. https://doi.org/10.1107/S0108767394005726.

(6) Sheldrick, G. M. *SADABS. Version 2.10. Bruker AXS Inc.*; Madison, WI, 2003.

(7) Sheldrick, G. M. A Short History of *SHELX*. *Acta Crystallogr., Sect. A: Found. Crystallogr.* **2008**, *64* (1), 112–122. https://doi.org/10.1107/S0108767307043930.

(8) Yap, W. T.; Doane, L. M. Determination of Diffusion Coefficients by Chronoamperometry with Unshielded Planar Stationary Electrodes. *Anal. Chem.* **1982**, *54* (8), 1437–1439. https://doi.org/10.1021/ac00245a041.
